# Mfap4: a promising target for enhanced liver regeneration and chronic liver disease treatment

**DOI:** 10.1038/s41536-023-00337-9

**Published:** 2023-11-07

**Authors:** Viktoriia Iakovleva, Anna Wuestefeld, Agnes Bee Leng Ong, Rong Gao, Neslihan Arife Kaya, May Yin Lee, Weiwei Zhai, Wai Leong Tam, Yock Young Dan, Torsten Wuestefeld

**Affiliations:** 1 Laboratory of In Vivo Genetics and Gene Therapy, Genome Institute of Singapore, Agency for Science, Technology and Research (A*STAR), 60 Biopolis Street, Singapore, 138672 Republic of Singapore; 2 Department of Medicine, Yong Loo Lin School of Medicine, National University of Singapore, Singapore, 119228 Republic of Singapore; 3 Laboratory of Translational Cancer Biology, Genome Institute of Singapore, Agency for Science, Technology and Research (A*STAR), 60 Biopolis Street, Singapore, 138672 Republic of Singapore; 4 Key Laboratory of Zoological Systematics and Evolution, Institute of Zoology, Chinese Academy of Sciences, 100101 Beijing, China; 5 Center for Excellence in Animal Evolution and Genetics, Chinese Academy of Sciences, Kunming, 650223 China; 6 Cancer Science Institute of Singapore, National University of Singapore, Singapore, 117599 Republic of Singapore; 7 Division of Gastroenterology and Hepatology, National University Health System, Singapore, 119074 Republic of Singapore; 8 School of Biological Science, Nanyang University of Singapore, Singapore, 637551 Republic of Singapore; 9 National Cancer Centre, Singapore, 169610 Republic of Singapore; 10 Present Address: Cancer Science Institute of Singapore, National University of Singapore, Singapore, 117599 Republic of Singapore

**Keywords:** RNAi, Non-alcoholic fatty liver disease, Liver fibrosis

## Abstract

The liver has a remarkable regenerative capacity. Nevertheless, under chronic liver-damaging conditions, this capacity becomes exhausted, allowing the accumulation of fibrotic tissue and leading to end-stage liver disease. Enhancing the endogenous regenerative capacity by targeting regeneration breaks is an innovative therapeutic approach. We set up an in vivo functional genetic screen to identify such regeneration breaks. As the top hit, we identified Microfibril associated protein 4 (Mfap4). Knockdown of Mfap4 in hepatocytes enhances cell proliferation, accelerates liver regeneration, and attenuates chronic liver disease by reducing liver fibrosis. Targeting Mfap4 modulates several liver regeneration-related pathways including mTOR. Our research opens the way to siRNA-based therapeutics to enhance hepatocyte-based liver regeneration.

## Introduction

The rising incidence of acute and chronic liver failure, which causes ~2 million deaths per year worldwide (World Health Organization, 2018, May 24), represents a major global health concern. Furthermore, liver disease is the only major cause of death still increasing year-on-year (British Liver Trust, 2018). The main drivers for end-stage liver disease are drug- and alcohol-induced liver damage, hepatitis virus infections (especially hepatitis B and C), and non-alcoholic fatty liver disease (or NAFLD/ NASH)^1^. Despite the breakthroughs in treating and preventing viral inflammations in the liver (vaccination against hepatitis B and hepatitis C combination therapies), the amount of people with end-stage liver disease is increasing, mostly due to the obesity crisis and aging society^2^. Therefore, there is a high demand for new therapeutic strategies and treatments for liver diseases.

Currently, liver transplantation is the only curative method to treat end-stage liver disease, however, the demand outnumbers by far the donor organ supply. Moreover, end-stage liver disease patients are often unfit to undergo major surgery^3^. Therefore, there is an urgent unmet medical need to develop new approaches to suppress disease progression and reverse end-stage liver disease.

The liver is the only visceral organ that possesses the remarkable capacity to regenerate. The ability of the liver to regenerate is central to liver homeostasis. Because the liver is the main site of drug detoxification, it is exposed to many toxic insults. Furthermore, through enterohepatic circulation, it is exposed to microbiota-related metabolites, which can trigger inflammation. Therefore, the liver is evolutionarily formed to regenerate damaged tissue rapidly, thereby preventing functional failure. It is known that as little as 25% of the original liver mass can regenerate back to its full size. Adult hepatocytes, representing the major cell type of the liver, are long-lived and normally do not undergo cell division, however, upon liver damage, they have the ability to enter the cell cycle and proliferate^4–6^. Serial transplantation experiments even indicate that hepatocytes have the capacity for nearly infinite proliferation^7^. Despite this amazing ability, the regenerative capacity of the liver seems limited, especially under chronic damaging conditions, leading to hepatocyte death and injuries^8,9^. Enhancing liver regeneration has become an important area of research for liver-related therapy as it can help to speed up the recovery of liver function and reduce the severity of liver disease. It was recently shown that the expression of the Yamanaka factors in the liver can help to enhance liver regeneration^10^ and that the transient expression of the major hepatic mitogens hepatocyte-growth factor (HGF) and epidermal-growth-factor (EGF) can reverse steatosis and accelerate restoration of liver function^11,12^.

Here we conducted an unbiased, hepatocyte centric in vivo functional genetic RNA interference^13^ (RNAi) screen in a very aggressive mouse model of chronic liver disease for the identification of new therapeutic targets for hepatic regenerative medicine. In our RNAi screen, four independent shRNAs targeting *Mfap4* were enriched, making Mfap4 our top target. The biological function of Mfap4 in the liver, especially in hepatocytes, is poorly understood. Mfap4 was described as a potential serum biomarker for liver fibrosis^14–16^ and strong staining for Mfap4 can be found in fibrotic scar tissue in the liver. However, the impact of *Mfap4* expression in hepatocytes in liver disease is unknown. We validated Mfap4 as a bona fide therapeutic target using a combination of in vitro and in vivo models. Knockdown of *Mfap4* enhanced proliferation of immortalized mouse and human hepatocytes. It also accelerated liver repopulation and liver regeneration after partial hepatectomy. Most importantly, it reduced fibrosis in the thioacetamide-triggered chronic liver damage model as well as in the “Western Diet” induced Non-Alcoholic Steatohepatitis (NASH) model. In-depth inter-species transcriptomic analysis and validation pinpoints to modulation of the mTOR pathway as an important mediator of the observed effect. The mTOR pathway is linked to liver regeneration, as it plays a crucial role in regulating cellular growth, proliferation, and metabolism in the liver. Our screening approach can be further expanded and allows genetic in vivo high-throughput screening for therapeutic targets. As our screening is hepatocyte centric, a fast translation to GalNAc-siRNA therapeutics is possible.

## Results

### High-throughput in vivo functional genetics identifies Mfap4 as a potential therapeutic target for chronic liver disease

**Fig. 1:**
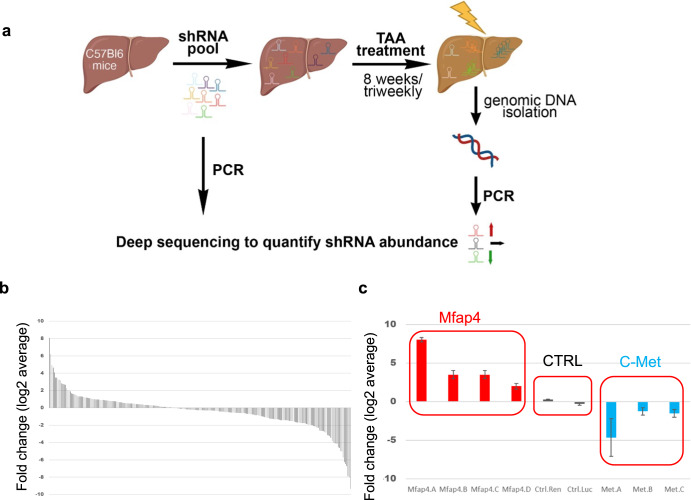
Functional genetic in vivo RNAi screen to identify therapeutic targets to enhance liver regeneration. **a** Outline of the screen. mice were injected with a pool of shRNAs (5 independent mice). After stable integration (~10% of hepatocytes), mice were treated with thioacetamide (TAA) to induce chronic liver damage associated with advanced liver fibrosis. Changes in shRNA abundance are detected by deep sequencing. This figure was created by the author V.I. with BioRender.com. **b** Representation of log2 fold change for each shRNA (ROMAampl library, 253 shRNAs, shown is the average value of 5 animals). **c** Functional genetic screen identifies Mfap4 as high confidence candidate (left red square) (zoom-in of (**b**) is shown). Several independent shRNAs were enriched targeting Mfap4 (left red square). Furthermore, non-targeting control (shNC) shRNAs (middle red square) did not show significant enrichment or depletion, and shRNAs targeting c-Met, an essential receptor for liver regeneration, are depleted (right red square).

An in vivo shRNA screen was conducted in a mouse model of chronic liver disease to identify new therapeutic targets to enhance the endogenous regenerative capacity of hepatocytes. The screened focused shRNA library is targeting genes located within recurrent focal genomic amplifications found in ~100 human HCCs^17,18^. We hypothesized, that using this library we could find a shRNA that enhances regeneration and at the same time is unlikely to trigger the transformation of the cells. The shRNA pool was subcloned into the p/T-RFP transposon vector and hydrodynamically co-delivered with an SB13 transposase encoding vector into the livers of C57BL/6JInv mice. The used delivery method leads to a hepatocyte-specific transduction^19–21^ and generates a chimeric mouse liver, where about 5–10% of hepatocytes have stable integration of an shRNA expressing construct^17,22^. Chronic liver damage was triggered by thioacetamide 3 times per week for 8 weeks^23–25^. Cycles of liver damage and compensatory regeneration induce a competitive environment. If the knockdown by a certain shRNA enhances endogenous regeneration or gives an advantage to hepatocytes in this environment, the cells can expand and enrichment for the shRNA can be detected. In contrast, if the expression of an shRNA is detrimental, this shRNA is depleted. No change compared to the starting pool indicates no effect by that shRNA. The abundance of the shRNAs was determined by Illumina-based deep sequencing. For sequencing, the genomic DNA was isolated from the whole liver to avoid any potential liver lobe-related bias, the shRNA expressing cassette was amplified with primers including Illumina adapter sequences, and the product was directly sequenced (Fig. 1a). Changes in abundance were calculated based on relative reads found in the liver compared to relative reads found in the starting library pool.

While the majority of shRNAs were depleted, a subset of shRNAs was highly enriched (Fig. 1b). We prioritized shRNAs, which were consistently enriched between all biological replicates (5 independent mice), as can be seen in the heatmap (Supplementary Fig. 1). Importantly and giving confidence in the screen, two independent non-targeting control shRNAs were not enriched or depleted. Furthermore, three independent shRNAs targeting c-MET the receptor for hepatocyte growth factor and essential for liver regeneration^26^ were depleted (Fig. 1c). To avoid following potential off-target effects of shRNAs, we focused on targets against which at least two independent shRNAs were enriched. Four independent shRNAs were found enriched targeting microfibril associated protein 4 (*Mfap4*), making it our top hit (Fig. 1c). In addition, we identified several targets against which at least 2 independent shRNAs were enriched (Supplementary Fig. 1).

### Knockdown of *Mfap4* accelerates proliferation of immortalized hepatocytes

**Fig. 2:**
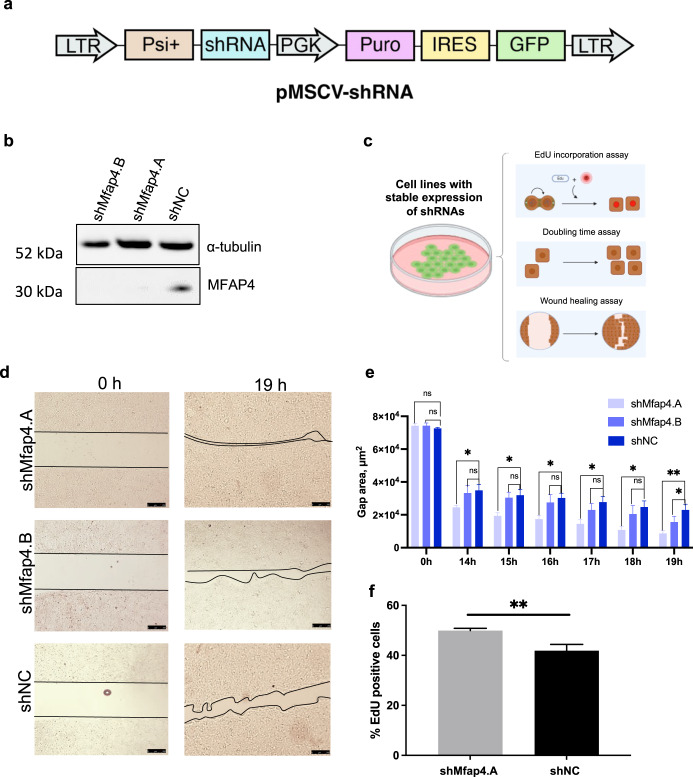
shRNA mediated knockdown of Mfap4 accelerates proliferation of liver cells. **a** Schematic representation of retroviral vector for generating cell lines with stable expression of GFP marker and shRNAs. **b** Western blot showing efficient knockdown of Mfap4 by the two top-enriched shRNAs from the screen (Fig. 1c). **c** Schematic outline for in vitro validation assays used in the study. **d** Wound healing assay. Cells with stable expression of shMfap4.A, shMfap4.B, or shNC respectively were grown to full confluence, then the silicon gasket was removed leaving a defined cell-free area (0 h time point). The filling of this “wound” gap was monitored. Representative images for each group are shown. Three technical replicates were performed. **e** Quantification of (**d**) over different time points is shown (Data were analyzed by ImageJ software; values of wound area in μm^2^ ± SEM; *p < 0.05, **p < 0.01, *n* = 3). **f** DNA synthesis of BNL CL.2 cells with stable expression of shMfap4.A or shNC was assessed by EdU incorporation. Shown is the value of % EdU positive cells ± SEM (**p < 0.01, 3 independent replicates).

Based on our hypothesis we expect that the enriched shRNAs accelerate the proliferation of hepatocytes. Therefore, we generated immortalized mouse hepatocyte cell lines with stable expression of the two top-enriched shRNAs targeting *Mfap4* or a non-coding control shRNA. First, we confirmed efficient on-target knockdown by these *Mfap4* targeting shRNAs (Fig. 2a, b). For further functional validation, we took advantage of these cell lines by running several proliferation assays (Fig. 2c). The knockdown of the two independent shRNAs targeting *Mfap4* accelerated significantly wound healing (Fig. 2d, e and Supplementary Fig. 2a, b). Furthermore, *Mfap4* knockdown leads to significantly higher EdU incorporation compared to the control, indicating faster cell replication (Fig. 2f). Consistent with these results it also shortens the cell doubling time (Supplementary Fig. 2c) and shows faster cell cycle progression (Supplementary Fig. 2d). Therefore, in line with our hypothesis, *Mfap4* knockdown enhances the endogenous proliferation rate of hepatocytes.

### *Mfap4* knockdown accelerates liver regeneration in vivo

**Fig. 3:**
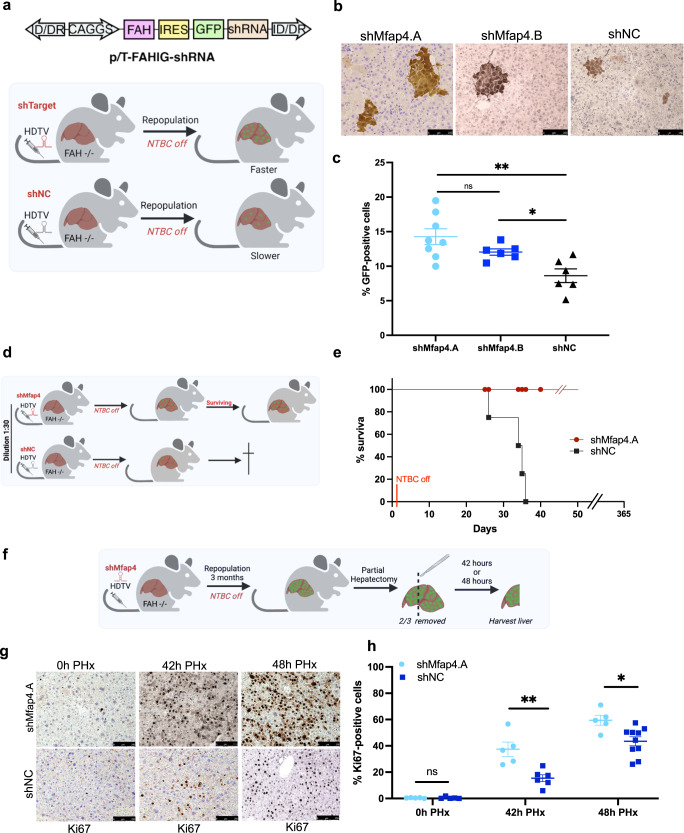
Mfap4 knockdown accelerates hepatocytes proliferation and liver regeneration in vivo. **a** FAH knockout mice based liver repopulation assay. The upper panel shows the outline of the transposon-based vector for the expression of the enzyme FAH, the marker GFP, and the shRNA of interest. The lower panels show the outline and rationale for the assay. If the knockdown of a certain shRNA is able to enhance regeneration and accelerate hepatocyte proliferation, we should be able to see a faster clonal expansion of GFP-positive hepatocytes compared to the control. **b** GFP immunohistochemistry staining (IHC) of liver tissues from FAH−/− mice injected with either p/T-FAHIG-shMfap4.A (*n* = 8), p/T-FAHIG-shMfap4.B (*n* = 6) or p/T-FAHIG-shNC (*n* = 6) at a 1 to 20 dilution. The liver was harvested 18 days post-injection (×200 magnification, representative pictures are shown, bar = 250 μm). **c** Quantification of GFP-positive hepatocytes (corresponding to (**b**)). Value shows % GFP-positive cells. Each dot represents one animal (*p < 0.05, **p < 0.01, ns non-significant). **d** Schematic outline of repopulation experiment, similar to (**a**). A further dilution of the amount of injected plasmids reduces the number of hepatocytes with stable expression of FAH, GFP, and the shRNA of interest, so that the FAH-expressing hepatocytes cannot fast enough expand and compensate for FAH−/− hepatocyte loss. However, an shRNA-dependent acceleration of regeneration might be able to allow survival. **e** Kaplan–Meier survival curve of FAH−/− mice injected with a 1:30 dilution of either p/T-FAHIG-shMfap4.A (*n* = 5) or p/T-FAHIG-shNC (*n* = 5) and SB13 (*p* < 0.05). **f** Experimental outline for investigating PH-induced liver regeneration. FAH−/− mice were injected with p/T-FAHIG-shRNA and SB13. After full liver repopulation, 2/3 PH was performed. Livers were harvested at 42 and 48 h after PH. **g** Representative photographs of DAB Ki67-stained liver sections 42 h (*n* = 5 per experimental group, *n* = 6 per control group) and 48 h (*n* = 5 per experimental group, *n* = 10 per control group) post hepatectomy are shown (×200 magnification). **h** Quantification of Ki67-positive hepatocytes (corresponding to (**g**)). Shown are % Ki67-positive cells at 0, 42, 48 h after partial hepatectomy. Each point represents one animal, and data shows average ± SEM (*p < 0.05, **p < 0.01, ns non-significant).

Based on our encouraging in vitro results, we went on to test the impact of hepatocyte-specific *Mfap4* knockdown in vivo. For this, we took advantage of the fumarylacetoacetate hydrolase (FAH) knockout mouse model^27^. The lack of this enzyme leads to the accumulation of intermediary hepatotoxins, which induces liver damage, leading finally to liver failure. However, correction of the FAH gene defect in a subset of hepatocytes leads to a strong selection pressure for these FAH-expressing hepatocytes. This can be used to repopulate FAH−/− livers^27^. We delivered a transposon-based construct for the expression of the missing enzyme FAH, the marker GFP, and the shRNA of interest via hydrodynamic tail vein injections (HDTV) to the hepatocytes of FAH−/− mice^28,29^ (Fig. 3a). Through this approach, we get in about 10% of hepatocytes stable integration of the construct and thus correction of the FAH gene defect. From these cells starts the repopulation of the liver. Our hypothesis was if the knockdown of *Mfap4* enhances regeneration and proliferation a faster clonal expansion of GFP-positive hepatocytes should be observed (Fig. 3a). We first performed a repopulation assay with a dilution of our constructs as 1:20 to ensure that we can observe clonal expansions originating from single hepatocytes. During the repopulation phase, livers were harvested, and the number of GFP-positive cells was counted. In line with our hypothesis, a faster clonal expansion was found in the case of *Mfap4* knockdown by antibody-based staining against GFP on paraffin sections compared to shNC control (Fig. 3b). Quantification showed a significant increase in the number of GFP-positive hepatocytes in the experimental group (shMfap4.A and shMfap4.B) compared to the control group (shNC) (Fig. 3c).

We further used a higher dilution of our constructs, to follow the idea that we reach a point where the number of hepatocytes with stable integration is insufficient to compensate fast enough for the loss of FAH−/− hepatocytes. We anticipated that shMfap4-driven accelerated repopulation will give the animals a survival advantage (Fig. 3d). As expected, the mice injected at a 1:30 dilution with the construct for expressing FAH, GFP, and shMfap4 were able to survive. In contrast, mice injected with the construct for expressing shNC died around 1-month post-NTBC withdrawal (Fig. 3e).

Next, we investigated whether *Mfap4* knockdown accelerates liver regeneration after partial hepatectomy. Partial Hepatectomy (PH) is a well-established acute liver damage model to investigate liver regeneration. After the surgical removal of 2/3 of the liver, hepatocytes synchronically enter the cell cycle in response to tissue loss and divide until the original liver mass is restored^4,30–32^. In this study, we anticipated that livers with *Mfap4* knockdown would proliferate faster compared to the control.

Here, we performed PH on fully repopulated FAH−/− mouse livers after HDTV injections with constructs for the expression of FAH, GFP, and shMfap4 or shNC, together with SB13 (Fig. 3f). Full repopulation means that >95% of hepatocytes express the shRNA of interest. The process of full repopulation took 3 months and after that, we performed 2/3 PH. We observed full repopulation of the resected liver lobes by macroscopic GFP imaging and DAB (3,3’-diaminobenzidine) based immunohistochemical (IHC) staining against GFP of liver tissue slides (data not shown). Livers were collected at two different time points—42 h and 48 h after partial hepatectomy. A significantly higher number of Ki67-positive hepatocytes were detected in the case of *Mfap4* knockdown compared to the control (Fig. 3g, h), indicating faster liver regeneration.

Taken together, our data show that *Mfap4* knockdown accelerates hepatocyte proliferation, liver repopulation, and liver regeneration in vivo.

### *Mfap4* knockdown attenuates chronic liver damage related liver fibrosis in vivo

Based on the strong pro-regenerative effects of *Mfap4* knockdown in hepatocytes we went on to test if shMfap4-mediated enhanced regeneration can attenuate chronic liver damage. As we first identified *Mfap4* as a potential target to enhance liver regeneration by a screen using the TAA-induced chronic liver damage model, we tested the therapeutic potential in this model first.

**Fig. 4:**
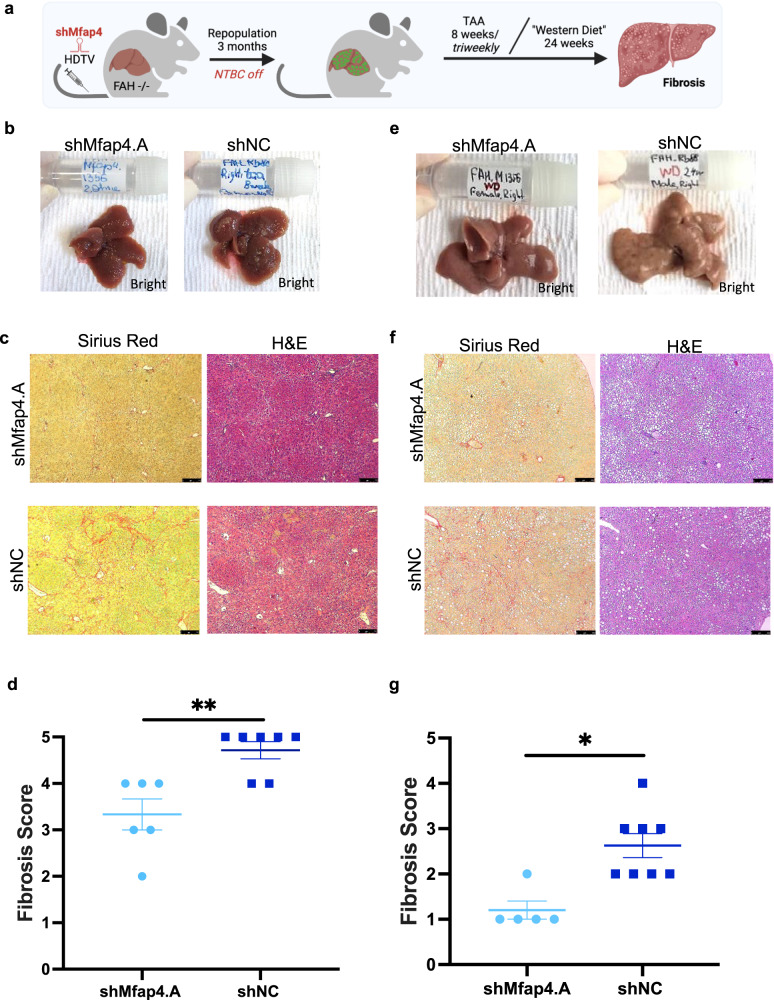
Mfap4 knockdown attenuates chronic liver damage related liver fibrosis. **a** Experimental outline of thioacetamide (TAA) induced chronic liver damage model and “Western Diet” induced NASH model. FAH−/− mice were injected with p/T-FAHIG-shRNA and SB13 constructs. After full liver repopulation, chronic liver damage was induced by repetitive doses of TAA administered intraperitoneal 3 times per week for 8 weeks or mice were exposed to the “Western Diet” (high-fat diet and 60 % fructose) for 24 weeks. Livers were harvested, processed, and analyzed. **b** Representative macro-photographs of the livers are shown (bright field; shMfap4 *n* = 6, shNC *n* = 7). Already macroscopic differences between groups were visible (TAA model). **c** Left column, Sirius red staining for fibrotic scar tissue of liver sections corresponding to (**b**). Right column H&E staining of liver sections corresponding to (**b**) (magnification ×50, representative images are shown, bar = 250 μm; *n* = 6 per experimental group and *n* = 7 per control group). **d** Quantification of (**c**). Fibrosis score is determined by a certified pathologist, who was blinded regarding the experimental group (each point represents one animal; **p < 0.01). **e** Representative macro-photographs of the livers are shown (bright field; shMfap4 *n* = 5, shNC, *n* = 8). Already macroscopic differences between groups were visible (“Western Diet” model). **f** Left column, Sirius red staining for fibrotic scar tissue of liver sections corresponding to (**e**). Right column H&E staining of liver sections corresponding to (**e**) (magnification ×50, representative images are shown, bar = 250 μm). **g** Quantification of (**f**). Fibrosis score is determined by a certified pathologist, who was blinded regarding the experimental group (each point represents one animal; **p < 0.01).

We repopulated the livers of FAH−/− mice so that >95% of hepatocytes express either shMfap4 or shNC. After the livers were fully repopulated, we induced chronic liver damage by repetitive injections of TAA for 8 weeks (Fig. 4a). After harvesting the livers we already saw macroscopic differences between the groups. Livers with *Mfap4* knockdown showed a smoother surface and had a healthier appearance (Fig. 4b). Consistent with our hypothesis, that enhanced liver regeneration can attenuate chronic liver disease, *Mfap4* knockdown attenuated TAA-induced liver damage and fibrosis. Tissue slides were stained for fibrotic scar tissue by Sirius red and liver morphology evaluation by H&E staining (Fig. 4c). H&E-stained liver tissues were evaluated by a certified pathologist, who also scored fibrosis in a blinded fashion. The pathology report confirmed a significant reduction in the fibrosis score of the experimental group compared to the control group (Fig. 4d), indicating the therapeutic potential of targeting *Mfap4* for chronic liver disease.

### *Mfap4* knockdown therapy: an innovative approach to treat non-alcoholic fatty liver disease (NAFLD)

As we saw the beneficial effects of targeting *Mfap4* in liver repopulation as well as in the very aggressive TAA model, we went on to test the impact of *Mfap4* knockdown in a physiological mouse model of NAFLD. The “Western diet” model (high-fat diet and 60% fructose in drinking water) has been recognized as one of the most relevant nutritional models reproducing features typical of the human NASH condition (hepatocyte cell death, ballooning, Mallory body formation, myeloid and lymphoid inflammatory infiltrate, and fibrosis)^33^. Non-alcoholic fatty liver disease (NAFLD) is one of the most common causes of chronic liver damage worldwide^34^. At least a quarter of the world’s population is affected by this disease and it is expected to be soon the number one reason for liver transplantations^35^. NAFLD is defined as when hepatocytes contain more than 5% of fat in the absence of any secondary cause such as viral hepatitis, alcohol excess, glycogen storage disorders, and endocrine abnormalities^36^. In its progressive form as Non-Alcoholic Steatohepatitis (NASH), it can lead to cirrhosis and liver cancer.

We used the “Western Diet” model to induce progressive NAFLD, leading to NASH and fibrosis (Fig. 4a). We performed a full repopulation of FAH−/− mouse livers; hence, all hepatocytes expressed either an shRNA targeting *Mfap4* or a non-targeting control shRNA. After full repopulation, the mice were exposed to the “Western Diet” for over 24 weeks (Fig. 4a). After harvesting the livers we already saw macroscopic differences between the groups. Livers with *Mfap4* knockdown showed a smoother surface and had a healthier appearance (Fig. 4e). Histopathological evaluation of Sirius red and H&E-stained tissue slides by a certified pathologist supports the therapeutic effect of *Mfap4* knockdown (Fig. 4f, g), as we saw significantly reduced fibrosis. In addition, we see a reduction in steatosis. Based on the macrovesicular steatosis score given by the certified pathologist in a blinded scoring, there is a reduction in the case of *Mfap4* knockdown (Supplementary Fig. 3a). This is consistent with oil red O staining of fat on liver tissue slides. Quantification of this staining shows reduced fat levels in the case of *Mfap4* knockdown (Supplementary Fig. 3b).

Moreover, *Mfap4* knockdown significantly reduced oval cell hyperplasia compared to the control group (Supplementary Fig. 3c). The oval cell population is a relatively small population of cells in the liver often found when the proliferation of hepatocytes is inhibited, followed by different types of hepatic injuries^37,38^. An increase in oval cells indicates the progression of the disease from mild to severer stage^39^. In contrast, the absence of oval cell hyperplasia indicates that hepatocytes are still able to compensate for damage and are not exhausted, as in the case of *Mfap4* knockdown, confirming the protective effect.

Taken together, our results show that the knockdown of *Mfap4* attenuates NASH progression by reducing liver fibrosis and steatosis. This effect is related to the increase in hepatocyte regenerative capacity, reflected in an absence of compensatory oval cell proliferation.

### Suppression of *MFAP4* enhances cell proliferation in human hepatocytes

**Fig. 5:**
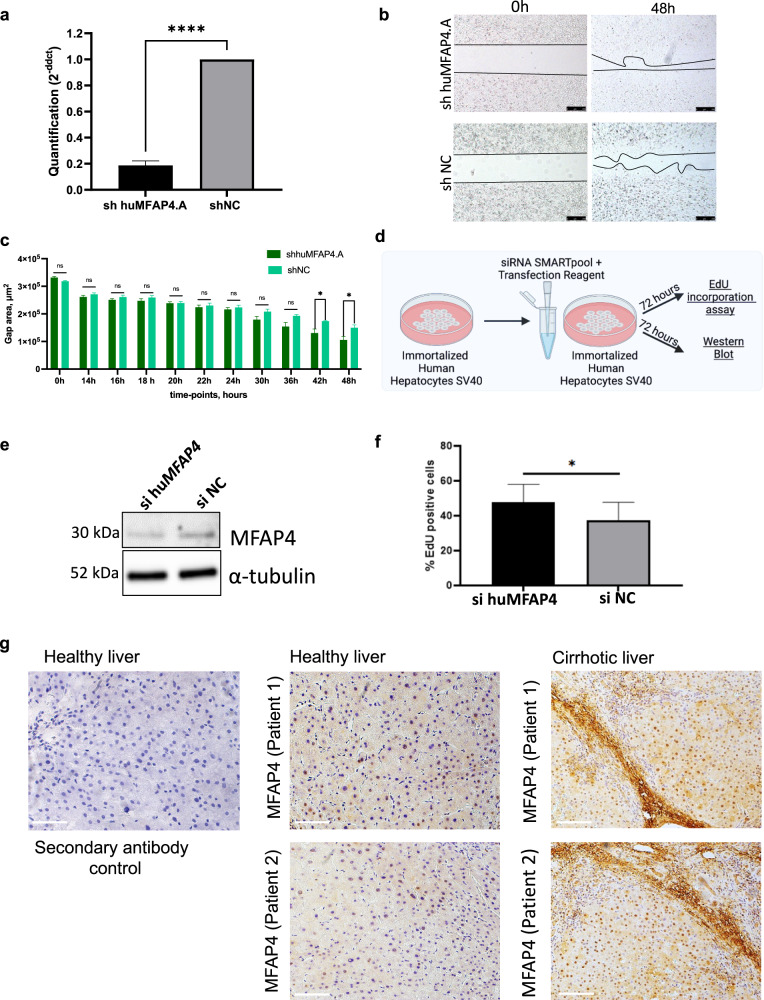
Effect of MFAP4 knockdown is conserved in humans. **a** qPCR showing efficient knockdown of human MFAP4 in immortalized human hepatocytes expressing shRNA targeting human MFAP4 (cells were generated by viral transfection, *p* < 0.0001). **b** Wound healing assay. Cells with stable expression of shhuMFAP4.A or shNC respectively were grown to full confluence, then the silicon gasket was removed leaving a defined cell-free area (0 h time point). The filling of this “wound” gap was monitored. Representative images for each group are shown. Three technical replicates were performed. **c** The quantification for (**b**) over different time points is shown (Data were analyzed by ImageJ software; values of wound area in μm^2^ ± SEM; *p < 0.05). **d** Schematic outline of the experiment. Immortalized hepatocytes are treated with siRNA for 72 h before harvesting cells for the isolation of RNA and proteins. **e** Western blot showing efficient knockdown of MFAP4 by siRNA SMARTPool (Dharmacon) in immortalized human hepatocytes. Cells were either treated with si huMFAP4 or siNC, α-Tubulin serves as a loading control (*n* = 3). **f** EdU incorporation assay (3 technical replicates). Shown is the value of % EdU positive cells ± SEM. Immortalized human hepatocytes were either treated with siRNA targeting human MFAP4 or siNC as control (*p < 0.05). **g** Human tissue samples from healthy and cirrhotic liver were stained against MFAP4 (DAB staining). On the left side, healthy liver tissue was stained without a primary antibody as a control. In the middle, staining of healthy liver tissue. On the right side, staining of cirrhotic human liver tissue (scale bar = 100 μm).

Next, we investigated if the effect of accelerating hepatocyte proliferation by *MFAP4* suppression is conserved in humans. For this, we generated human hepatocytes with stable expression of either an shRNA targeting human *MFAP4* (shhuMFAP4.A) or a non-targeting control shRNA (shNC). Expression of the *MFAP*4 targeting shRNA leads to a strong knockdown of *MFAP4* in the human hepatocytes (Fig. 5a). We tested if this *MFAP4* knockdown in human cells accelerates wound healing. We found significantly accelerated wound healing as was also observed for mouse immortalized hepatocytes (Fig. 5b, c). This indicates that the effect of accelerated hepatocyte proliferation is conserved between mouse and man. In addition, we treated human hepatocytes with sihuMFAP4 SMARTPool and investigated the effect on proliferation (Fig. 5d). Treatment with sihuMFAP4 led to efficient transient knockdown of *MFAP4* (Fig. 5e). We tested if the transient knockdown is sufficient to induce accelerated proliferation of human hepatocytes. Checking for EdU incorporation we detected a significant increase in the case of *MFAP4* suppression (Fig. 5f). This suggests that using siRNA therapeutics targeting *MFAP4* in hepatocytes is a viable approach.

MFAP4 was suggested as a potential biomarker for the non-invasive assessment of hepatic fibrosis in hepatitis C patients^14^. In addition, previous studies showed increased MFAP4 staining in liver and lung fibrosis^15^. We performed staining against MFAP4 and detected a higher amount of MFAP4 in human cirrhotic liver tissue samples from NASH patients (Fig. 5g). Interestingly, besides strong staining in fibrotic scar areas as expected, MFAP4 was also detected in the cytoplasm and nucleus of hepatocytes in the cirrhotic liver (Fig. 5g). In line with this we observed higher expression of *MFAP4* in the cirrhotic patient samples compared to healthy controls (Supplementary Fig. 4a). Consistent with our findings we analyzed a publicly available dataset of 206 patients and found a fibrosis-stage dependent significant increase in MFAP4 expression (Supplementary Fig. 4b)^40^. Our data support the notion, that we can enhance human hepatocyte proliferation by targeting *MFAP4* and that *MFAP4* is dysregulated in human hepatocytes of diseased livers.

### The knockdown of *MFAP4* affects key pathways involved in liver regeneration

**Fig. 6:**
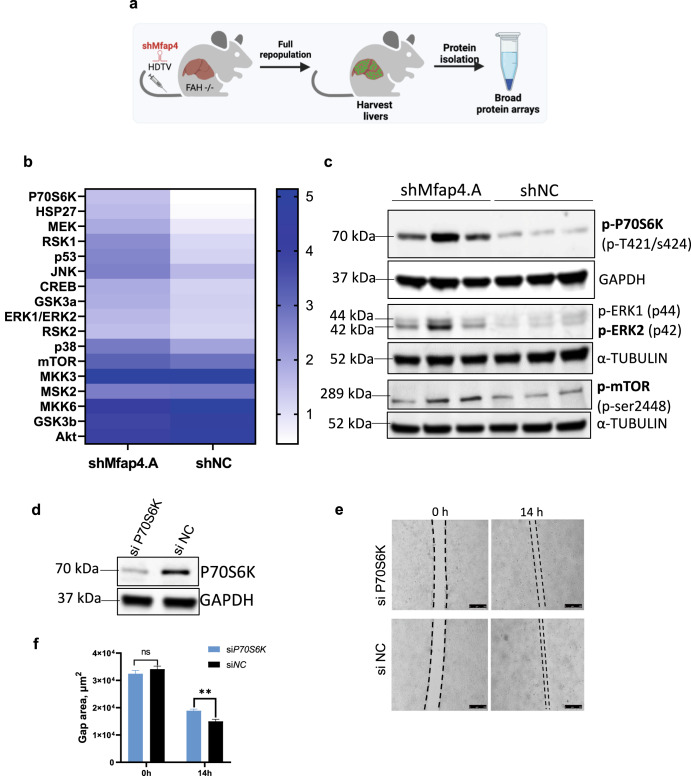
Knockdown of Mfap4 impacts mTOR and ERK signaling. **a** Schematic outline of the experiment. Whole-cell protein extracts from repopulated mouse livers were isolated and analyzed by protein array. **b** Heat map shows results for phosphorylation specific MAPK pathway protein array. Whole-cell protein extracts from repopulated mouse livers with stable expression of either shMfap4 or shNC were analyzed (shown is the relative signal intensity). **c** Western blot analysis for the phosphorylation status of P70S6k, ERK1,2, mTOR, corresponding to (**b**). α-Tubulin or GAPDH serves as a loading control (*n* = 3). **d** Knockdown test of P70S6K siRNA pool. Western blot of protein extracts from BNL.CL2 cells with stable shMfap4 expression were treated either with siP70S6k or siNC. GAPDH serves as a loading control (*n* = 3). **e** Wound healing under double knockdown conditions. Liver cell line with stable knockdown of Mfap4 was expanded, after that cells were treated with respective siRNAs, and the silicon gasket was removed. Wound healing was monitored. Slower growth and migration were observed in the case of double-knockdown of Mfap4 and p70S6k. **f** Quantification of (**e**) is shown (values of wound area in μm^2^ ± SEM; *n* = 3; **p < 0.01; ns non-significant).

As phosphorylation cascades are essential for liver regeneration but cannot be investigated by pure transcriptomics, we explored different pathways through phosphorylation-specific protein arrays and further validated data by Western Blot. First, we repopulated the livers of FAH−/− mice, so that nearly every hepatocyte expresses an shRNA against *Mfap4* or a control shNC. Whole-cell protein extracts from these livers were used for a broad pathway protein array (Fig. 6a). Interestingly, the top-upregulated hit was activated P70S6K. Phosphorylation and activation of P70S6K are essential for liver regeneration^41^. Furthermore, P70S6K is downstream of mTOR^42,43^, GSK3^44^, and ERK^45^ signaling pathways which are all stronger activated in case of *Mfap4* knockdown in the liver (Fig. 6b). Stronger activation of JNK and ERK as detected by the protein array is associated with hepatocyte proliferation^46^. Furthermore, impairments in P70S6K and ERK signaling are linked to the age-dependent decline of the liver’s regenerative capacity^47^. Consistent with the protein array data, we validated stronger activation of mTOR, ERK, and P70S6K by Western Blot using whole-cell extracts from fully repopulated livers (Fig. 6c). To prove the importance of P70S6K activation we performed a reverse rescue phenotype experiment. We conducted the in vitro wound-healing assay under double knockdown conditions. For this, we used our hepatocyte lines with stable expression of shMfap4, where we saw *Mfap4* knockdown-dependent faster wound closure. We treated these cell lines with a pool of siRNAs targeting P70S6K or a control siNC (Fig. 6d–f). We found that the knockdown of P70S6K abolishes accelerated wound healing. This indicates that *Mfap4* knockdown-mediated accelerated wound healing depends on the activation of P70S6K. We further investigated if upstream inhibition of mTOR using the mTOR inhibitor TORIN1 treatment shows a similar effect on wound healing (Supplementary Fig. 5a, b). In line with our results regarding P70S6K knockdown, mTOR inhibition strongly attenuates *Mfap4* knockdown driven faster wound healing. In summary, suppression of *Mfap4* in hepatocytes affects key liver regeneration pathways.

**Fig. 7:**
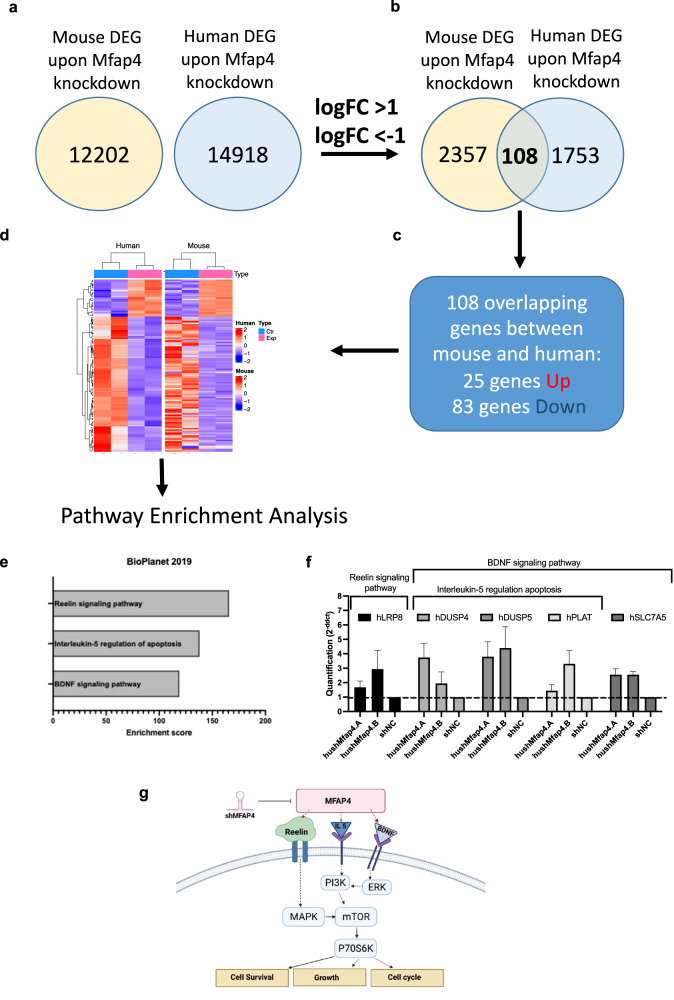
The integrative transcriptomic analysis highlights Mfap4 knockdown dependent affected pathways, which are conserved between mice and man. **a** Number of identified differentially expressed genes in immortalized mouse hepatocytes with Mfap4 knockdown and immortalized human hepatocytes with MFAP4 knockdown. **b** Filtered DEG from (**a**) with logFC >1 or logFC < −1 showed 108 overlapping genes between mouse and human. **c** Among 108 overlapped genes, 25 genes were upregulated, 83 genes were downregulated. **d** Heatmap showed overlapping up- and downregulated genes. **e** Pathway analysis of overlapping DEG highlights the Reelin signaling pathway, IL-5 regulation of apoptosis, and the BDNF signaling pathway. **f** Validation of upregulated genes indicated by pathway analysis (qPCR). **g** Proposed mechanism of action upon Mfap4 knockdown. This figure was created by the authors V.I. and N.A.K. with BioRender.com.

To further understand the underlying mechanism of *MFAP4* knockdown-mediated enhanced regeneration, we went on and conducted in-depth inter-species transcriptomic profiling of immortalized human and mouse hepatocytes with stable Mfap4 knockdown and respective controls. Differentially expressed genes were identified by comparing relative gene expression changes in the case of Mfap4 knockdown (by two independent shRNA targeting human or mouse Mfap4) compared to the non-targeting control (shNC). The correlation map (Supplementary Fig. 6a, b) showed a strong association between the experimental group and the control group. PCA analysis revealed similarities between the experimental and control group and the formation of clusters for experimental samples as well as for non-targeting control samples (Supplementary Fig. 6c, d). Volcano plots showed the distribution of upregulated and downregulated genes in both mouse and human immortalized hepatocyte cell lines (Supplementary Fig. 6e, f). We focused on Mfap4 knockdown-driven differential expressed genes (DEGs), which overlap between both species (Fig. 7). As suppression of Mfap4 in both human and mouse hepatocytes accelerate hepatocyte proliferation, such DEGs should represent the conserved signature for understanding the underlying pathways. We identified a subset of 108 genes that overlapped (Fig. 7a–d), 25 were significantly up- and 83 downregulated (cut of log2 fold-change >1 or <−1, *p* value < 0.05). These DEGs were further parsed through Enrichr (Biolanet 2019) database^48–50^ to identify distinct but overlapping pathways upon *MFAP4* knockdown. The BDNF signaling pathway, Interleukin-5 regulation of apoptosis pathway, and the Reelin signaling pathway were significantly upregulated upon *MFAP4* knockdown (Fig. 7e). Validation by qPCR analysis for genes that were identified through the Enrichr algorithm and linked to the pathways was conducted. qPCR analysis confirmed the upregulation of human LRP8, human DUSP4, human DUSP5, human PLAT, and human SLC7A5 genes (Fig. 7f). While the Reelin pathway is mainly explored in the brain, it also triggers core pathways associated with liver regeneration and biology such as PI3K-Akt signaling, and impacts notch and N-Cadherin signaling^51,52^. Reelin also activates the mTOR-S6K1 pathway^51^. In addition, BDNF singling pathway leads to the activation of mTOR and its downstream target P70S6K^53^, which is important for cell survival and cell growth^54^. This connects our inter-species transcriptome analysis with our protein array analysis and highlights the impact of inhibition of *MFAP4* on liver regeneration-related pathways. Therefore, we propose the following mechanism. Knockdown of *MFAP4* triggers activation of the mTOR pathway, priming the hepatocytes for proliferation upon liver damage (Fig. 7g).

### Chronic *Mfap4* knockdown in hepatocytes does not compromise liver integrity or function

**Fig. 8:**
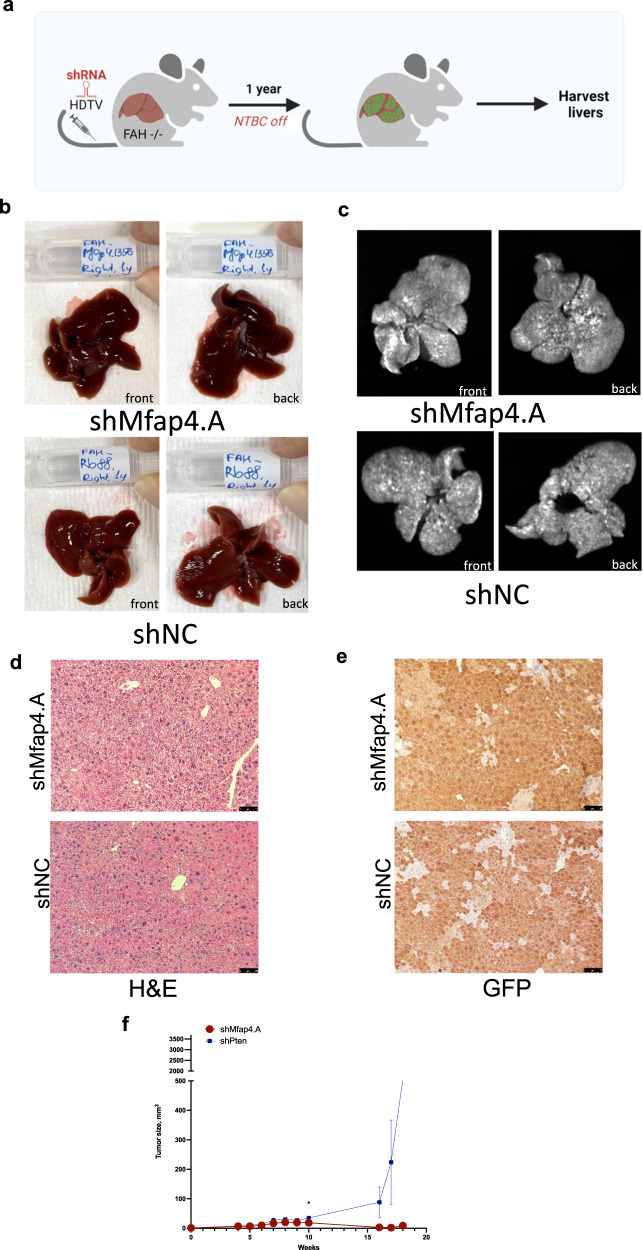
Chronic Mfap4 knockdown in hepatocytes does not compromise liver integrity or function. **a** Schematic representation of the experiment. Constructs were delivered by HDTV; then, FAH−/− mice were kept for 1 year to observe any tumor formation. **b** Bright field. Representative pictures for experiment and control are shown (both surface sides of the liver). No macroscopic tumors or liver architecture abnormalities were observed. **c** GFP-imaging. Representative pictures for experiment and control are shown. No macroscopic GFP-positive tumors were observed in experiment as well as in control. Livers are fully repopulated (strong GFP-positive signal). **d** Hematoxylin and eosin staining (scale bar is 500 µm, magnification is ×100). Representative pictures are shown. No malignant lesions were observed by a certified pathologist in both: experimental and control group. **e** GFP (DAB) staining indicates full liver repopulation after 1 year (representative pictures are shown; scale bar is 500 µm, and magnification is ×100). **f** Growth curves of subcutaneous tumors on NSG mice originating from the indicated subcutaneously injected cell lines stably transduced with shMfap4 or shPten (data are presented as mean ± SEM). No tumor growth was detected in case of Mfap4 knockout.

Increased proliferation is a known hallmark of malignant growth. Therefore, we investigated the risk of promoting tumorigenesis by long-term *Mfap4* knockdown. Fully repopulated, FAH−/− mice with stable intrahepatic *Mfap4* knockdown were monitored for 1 year (Fig. 8a). At the point of sacrifice, the mice looked healthy, and macroscopic pathological evaluation of the liver tissue did not show any signs of disease (Fig. 8b). None of the mice developed GFP-positive, shMfap4-expressing liver tumors (Fig. 8c). Furthermore, the histological evaluation of these livers did not show any abnormalities (Fig. 8d, e). Importantly, there is no difference in proliferating hepatocytes between both groups after 1 year repopulation (Supplementary Fig. 7a). Even more the knockdown of Mfap4 in human liver cancer cells (HepG2) did not accelerate cell proliferation further (Supplementary Fig. 7b). These results indicate that long-term knockdown of *Mfap4* in the liver is safe.

Additionally, we performed subcutaneous injections of 2*10^6^ AML cells with stable *Mfap4* knockdown or stable *Pten* knockdown into the flanks of NSG mice. After that, the sites of the injections were monitored for tumor development. As expected, *Mfap4* knockdown did not lead to tumor development, whereas *Pten* knockdown triggered the transformation of the cells leading to tumor formation (Fig. 8f). *Mfap4* suppression does not drive malignant transformation indicating that hepatocyte-specific siMfap4 treatment is safe.

## Discussion

In this study, we conducted an in vivo functional RNAi screen and identified Mfap4 as a potential target for enhancing liver regeneration. We confirmed through validation experiments, that knockdown of Mfap4 in mouse and human hepatocytes accelerates the proliferation of these cells. We showed that the knockdown of *Mfap4* in hepatocytes in vivo enhances liver regeneration as well as liver repopulation and attenuates chronic liver disease using two different mouse models. Knockdown of *MFAP4* in hepatocytes influences several liver regeneration-related pathways. This mechanism is conserved between mouse and man. In summary, we validated *MFAP4* as a therapeutic target for enhancing liver regeneration and attenuating chronic liver disease.

The liver has a unique regenerative capacity. It is known that as little as 25% of the original liver mass is sufficient and able to regenerate to the original liver amount after liver damage. Serial transplantation experiments indicate a nearly infinite proliferation capacity of hepatocytes^7^. However, the regenerative capacity of the liver seems limited under chronic damaging conditions^8^ and declines during aging^55,56^. Interestingly, the progression to cirrhosis takes longer if hepatitis C infection occurred in younger individuals than in older individuals^57^. This also holds the hope, that by enhancing the endogenous regenerative capacity of the liver we can delay liver function failure and cirrhosis.

Through in vivo functional genetics and genomics, we can expand the number of therapeutic targets. Interestingly, MFAP4 was described as a potential biomarker for liver and lung fibrosis before^14–16^. In these reports, the emphasis was on the high amount of MFAP4 found in fibrotic scar tissue. We show that targeting Mfap4 in the hepatocyte can enhance the regenerative capacity of hepatocytes and counteract chronic disease. Interestingly targeting *MFAP4* seems to enhance the regenerative power beyond the liver. In recent reports knockout of Mfap4 protected against renal fibrosis^58^ and left ventricular remodeling and dysfunction in heart failure^59,60^. In the context of these studies and our data, targeting *MFAP4* might be a therapeutic approach to a multitude of fibrotic diseases. It would be interesting to evaluate if the beneficial effect in these organs is also linked to a better regenerative response.

For any pro-regenerative therapy, which enhances cell proliferation, the potential for driving cancer is a key concern. Therefore, we evaluated this very carefully. *Mfap4* knockdown did not lead to hepatomegaly upon liver damage, indicating a controlled regenerative process. Furthermore, knockdown of *MFAP4* in human liver cancer cells does not accelerate their proliferation further and knockdown of *Mfap4* in immortalized, sensitized hepatocytes did not transform these cells. Even after 1 year of shMfap4 expression we did not see higher proliferation of hepatocytes compared to the control. Therefore we think targeting Mfap4 shows a favorable safety profile. We hypothesized that the knockdown of *Mfap4* releases the break from liver regeneration, but additional signals are needed to start the engine.

Targeting *MFAP4* enhances liver regeneration and counteracts chronic liver disease. Thus, the translation of our findings into the clinic can directly help patients with chronic liver disease and reduce health costs. Especially in the context of RNAi-based therapeutics, the translation can be efficient and quick.

## Methods

### Animals

For in vivo studies, C57BL/6JInv mice were purchased from InVivos (Singapore), which is a commercial licensee of The Jackson Laboratory (JAX®). FAH knockout mice have been generated by Dr. Markus Grompe^61^ (Oregon Health Sciences University) and were obtained in the C57BL/6 background from Dr. Arndt Vogel (Hannover Medical School). The NTBC drug (2-[2-nitro-4-(trifluoromethyl)benzoyl]cyclohexane-1,3-dione) was given to FAH−/− mice at 19.2 mg per 1-liter drinking water to prevent liver failure.

Mice were of mixed genders. Mice within experiments were 6–8 weeks old and housed under specific pathogen-free conditions in accordance with institutional guidelines of the Biological Recourse Center (BRC, A*Star). All animal experiments have been approved by the Institutional Animal Care and Use Committee (A*Star, IACUC No. 191452, Singapore).

### Human subjects

Four human samples were collected and analyzed in collaboration with Dr. Yock Young DAN (NUHS, Singapore). All samples were obtained under protocols approved by the Ethics Committee of the National University Hospital, Singapore, and informed consent was obtained from each patient. IRB#: NHG DSRB Ref:2016/00580. Study title: Ensemble of Multi-disciplinary Systems and Integrated Omics for NAFLD (EMULSION) diagnostic and therapeutic discovery. Sub-study: Phenotypic-genomic profiling of Asian Non-Alcoholic Fatty Liver Disease to identify mechanistic pathways as potential targets for intervention.

### Human and mouse cell lines

Phoenix, BNL CL.2, and AML12 were purchased from ATCC (www.atcc.org; CRL-3213, TIB73, and CRL-2254 retrospectively). Immortalized human hepatocytes-SV40 were purchased from Creative Bioarray (Cat# CSC-I9016L).

### shRNA library cloning, shRNA cloning, vector construction

The shRNAs from the ROMA-amplicon library^17^ were cloned into our pGAGGS-RFP-mir30-5’ transposon backbone. The shRNA library was excised from the original MSCV-based retroviral vector within the miR30 flanking regions using the restriction sites XhoI and MluI/AscI. After the cloning procedure, deep sequencing of the transposon-based library ensured full representation of all shRNAs. Individual shRNAs for validation experiments were synthesized as 97 bp oligos. PCR-amplified and cloned into pGAGGS-RFP-mir30, and verified by sequencing. C57Bl6 wild-type mice (5 mice) were injected by hydrodynamic tail vein injections (HDTV), and after 5 days, thioacetamide (TAA) treatment was started (0.2 mg TAA per g body weight, three times per week for 8 weeks). After the last injection of TAA, the mice were given a 1-week recovery period, and then the livers were harvested. Genomic DNA from the whole liver of each mouse was isolated. Then, the shRNA cassettes were PCR amplified using an established protocol. The primer for the PCR amplification step included the Illumina adapter sequences and a sample identifier (barcode 8 bp) so that Illumina HiSeq 4000 System could directly sequence the purified PCR products. In this case, all samples with different barcodes were mixed and run on a single flow cell. The GIS (A*Star) bioinformatics core support did the first deconvolution of deep sequencing data to generate lists of detected shRNAs and their number of reads for each sample. Then, we further compared the shRNA distribution to the starting pool.

Individual shRNAs for validation experiments in vitro were designed based on shRNAs from the ROMA-amplicon library screen and synthesized as 97 bp oligos DNA (IDT, Integrated DNA Technologies, Singapore). Individual human shRNAs for validation experiments in vitro were designed using the DSIR tool^62^. The shRNAs were PCR cloned into MSCV.

For experiments in vivo, transposable elements for stable coexpression of FAH, green fluorescent protein (GFP), and microRNA-based shRNAs were generated according to the approach which was described previously^63^.

### shRNA recovery, identification, determination of representation

Genomic DNA was isolated from liver tissues as indicated, and the integrated transposon sequences were amplified using primers^64^ flanking the miR30 cassette harboring the Illumina adapter sequence. The same PCR approach was applied to the library pool plasmid DNA. Deep sequencing analyses were done using the Illumina-HiSeq 4000 System as described previously. The GIS (A*Star) bioinformatics core support did the first deconvolution of deep sequencing data to generate lists of detected shRNAs and their number of reads for each sample. The sequences were aligned to the shRNA library data (only 100% matches were allowed) and summarized.

We combined the data from five individually sequenced mice and compared the mean changes. shRNAs with at least two-fold change (positive or negative regulation) were selected for further analysis. For targets for further validation, we looked for at least two independent shRNAs scoring. Sequence of shRNAs listed in Supplementary Table 2.

### In vitro knockdown tests

Phoenix packaging cells were used for the production of retroviral particles. The Phoenix cells were transfected with a retroviral vector via calcium phosphate–mediated transfection. Twenty-four hours later, viral supernatant was applied directly to BNL CL.2 and AML12 cells. Polybrene was added (1–10 μg/ml) to enhance infection efficiency. Target cells were selected using puromycin (1–10 μg/ml) and expanded or harvested for the preparation of whole-cell protein extracts or isolation of RNA.

### Wound healing assay

For the wound healing assay, cells were seeded in 2-well µ-dishes (Ibidi Technologies, Cat # 81176). These dishes include a silicon gasket which generates a defined 500um cell-free gap. Stable cell lines with Mfap4 knockdown were generated and then, incubated in a fresh DMEM (GIBCO) medium for 24 h. After that, the silicon gasket was removed once the cells grew with 100% confluence within the well. The two opposite cell fronts generated by the 500 um gap were observed under a microscope at 0, 14, 15, 16, 17, 18, 19, 20, 21, 22, 23, and 24 h time points. The gap represents a wound, and over time cells will proliferate and fill this “wound”.

For wound healing assay with mTOR inhibitor (Supplementary Fig. 5) we used the Torin1 compound (MedChem Express, cat# HY-13003). We dissolved Torin1 in DMSO according to the manufacturer’s protocol and prepared a 1 mM stock solution. For the wound healing experiment, cells were seeded in 2-well µ-dishes. In 4 h, Torin1 was added in three different concentrations: 0.1, 0.3, and 0.5 µM. In 24 h, the silicon gasket was removed, and wound closure was observed during the next 24 h.

### EdU incorporation assay

For the EdU proliferation assay, we used Click-iT™ EdU Cell Proliferation Kit for Imaging Alexa Fluor™ 594 dye from Thermo Fisher (Cat # C10339). EdU incorporation assay identifies DNA synthesis. Cell proliferation is linked to de novo DNA synthesis during the S-phase of the cell cycle. We used mouse liver cell line BNL CL.2 and immortalized human hepatocytes-SV40. The procedures were done according to the manufacturer’s instructions. A stable BNL CL.2 cell line with Mfap4 knockdown was created, and immortalized human hepatocytes after siRNA transfection were used for the assay.

### Cell cycle assay

For this assay, we used Muse Cell Cycle Kit (Luminex, Cat # MCH100106). Cells were seeded at the same seeding densities at 0 h time-point. Then, a one-time 100 µl Thymidine (200 mM) was added for the cell cycle synchronization. Within 12 h, cells were washed with PBS, and the new portion of DMEM (GIBCO) was added. After that, cells grew for 24 h and were harvested. Next, cell samples were fixed with 70% ice-cold ethanol. The staining protocol was performed according to manufacturer instructions. The cell cycle was analyzed using Guava Muse Cell Analyzer (Luminex Muse/Biomed Diagnostics). The Muse Cell Cycle Software Module performed calculations automatically. We use mouse liver cell line BNL CL.2 with shRNAs against Mfap4 or non-coding control shRNA.

### Doubling time assay

Cells were seeded at the same seeding densities at 0 h time-point. Then, one-time 100 µl Thymidine (200 mM) was added at 12 h time-point and cells were kept overnight at 37 °C, 5% CO_2_. Thymidine is a DNA synthesis inhibitor and is used to synchronize the cells. The following day, cells were washed in DPBS twice, and 10 h later, the first measurement of cell number was taken at different time points: 24, 48, and 72 h.

### siRNA transfection

ON-TARGETplus Human MFAP4 siRNA SMARTpool (a mixture of four different siRNAs at a ratio of 1:1:1:1; 5 nmol) was purchased from Dharmacon and dissolved in siRNA buffer (Dharmacon, catalog # B-002000-UB-100). Immortalized human hepatocytes-SV40 were seeded at the same densities on 6-weel collagen plates (Thermo Fisher, cat# A1142801), and incubated in SuperCult® Immortalized HumanHepatocyte Growth Medium (Creative Bioarray, cat# CSC-C9441L), and kept at 37 °C, 5% CO_2_. siRNA transfection was performed according to manufacturer protocol. Sequence of siRNAs listed in Supplementary Table 2.

### Hydrodynamic tail vein injections (HDTV**)**

Vectors for hydrodynamic tail vein injection^29^ were prepared using the Qiagen EndoFreeMaxi Kit (Qiagen, Hilden, Germany). For transposon-mediated gene transfer, animals received a 5:1 molar ratio of the transposon to transposase-encoding plasmid (25 mg total DNA, unless otherwise stated; 5 mg transposase sleeping beauty 13). DNA was suspended in saline solution at a final volume of 10% of the animal’s body weight and injected in less than 10 s via the tail vein.

Progression of liver repopulation was monitored at different time points by whole-liver GFP imaging using the Syngene system (UK).

### Repopulation assays

We performed two repopulation assays with the different dilutions of our constructs. One dilution of our constructs was 1:20 (1.25 mg of plasmid together with 0.25 mg of transposase sleeping beauty 13), following the standard ratio of 5:1 (25 mg of plasmid and 5 mg of sleeping beauty 13 (SB13)).

To identify the survival rate under higher selection pressure, we performed a repopulation assay with the dilution of our constructs as 1:30 (0.83 mg of plasmid together with 0.17 mg of SB13) following the standard ratio 5:1 described above.

### Thioacetamide (TAA) administration

To induce chronic liver damage, thioacetamide (TAA, Sigma-Aldrich) was delivered by intraperitoneal injections (IP). Intraperitoneal administration of TAA was performed three times per week for 8 weeks with a concentration of 40 mg/ml. TAA was dissolved in saline solution. The final administrated concentration for each mouse is 200 mg/kg.

### Partial hepatectomy

Two-thirds (partial) hepatectomy was performed on fully repopulated FAH−/− mice as described before^31^. The median, right, and caudate liver lobes were surgically removed while mice were under general isoflurane anesthesia. After that, the remaining liver was collected at stated time points and further analyzed.

### Histology and Immunohistochemistry

Histopathological evaluation of murine livers was performed on hematoxylin and eosin (H&E) and picro sirius red (PSR) stained paraffin sections by board-certified pathologists (Advanced Molecular Pathology Laboratory (AMPL) IMCB, A*STAR, Singapore). Staining and immunohistochemistry for Ki67 (1:200, Abcam, Cat# ab15580) and GFP (1:100, Cell Signaling Technology, Cat# 2956) were performed on paraffin-embedded liver sections by AMPL. Microscopic analyses were performed using Observer Z1 microscope (Zeiss). Five high-power fields were counted on two liver sections from each mouse liver (200X, >200 counted cells per field).

### Western blotting

Whole-cell protein extracts were prepared from mouse (AML12, BNL Cl.2) cell lines and immortalized human hepatocytes-SV40. Whole-cell protein extracts from mouse livers were isolated to perform pathway analysis. Proteins were separated on a 10% or 4–20% gradient Mini-PROTEAN® TGX™ Precast Protein Gels (Bio-Red).

### Protein arrays

Whole-cell protein extracts were prepared from mouse tissue samples, and the protein array was done using MAPK pathway phosphorylation Array (RayBiotech, C1; cat# AAH-MAPK-1-8). The procedures were done according to the manufacturer’s instructions. The difference between experimental and control samples was identified, and then potential proteins were chosen for further western blotting validation.

### mRNA expression, quantitative PCR analysis

mRNA was isolated from whole cells or liver tissue using Isolate II RNA Mini Kit (Bioline). cDNA synthesis was done with qScript cDNA Synthesis Kit (Quanta, Cat# 95047-100). Quantitative qPCR was performed with PerfeCTa SYBRgreen Master Mix (Avantor, Cat# QUNT95072-012). Values were normalized toward human GAPDH quantification. Sequence of primers used for qPCR analyses listed in Supplementary Table 1.

### Gene expression analysis

Differentially expressed genes were identified separately for human and mouse samples using the “DESeq2” R package. Genes with a mean raw count of less than ten across samples were filtered out before the analysis. An adjusted *p* value threshold of less than 0.1 and a log2 fold change of greater than 1 or less than −1 was used to define up-regulated or downregulated genes respectively in both human and mouse data. Human orthologs of mouse genes were obtained using the “biomaRt” R package by obtaining the “hgnc symbol” corresponding to the “mgi symbol” of genes in the mouse data with getLDS() function. Overlapping differentially expressed genes are defined as genes with a corresponding ortholog in another dataset after *p* value and fold change filtering.

### Quantification and statistical analysis

The cumulative survival of mice was assessed by Kaplan–Meier analysis, and statistical significance was calculated using the log-rank test. Unless otherwise stated, for all other comparisons, statistical significance was calculated using the unpaired two-tailed Student’s *t* test. *p* values < 0.05 were considered to indicate statistical significance (“*” equal *p* ≤ 0.05, “**” equal *p* ≤ 0.01, “***” equal *p* ≤ 0.001). All statistical analyses were performed using GraphPad Prism 9 software.

### Supplementary information

Supplementary_Material

Reporting-summary

## Data Availability

Stable cell lines were generated in the Laboratory of In Vivo Genetics and Gene Therapy, Genome Institute of Singapore (GIS, A*STAR) and are available upon request. Microscopy data reported in this paper will be shared by the lead contact upon request.

## Resource availability

Requests for information and reagents should be directed to and will be fulfilled by the lead contact, T.W. (wustefeldt@gis.a-star.edu.sg).

## Materials availability

All unique/stable reagents and data generated in this study are available from the lead contact without restriction.

## Code availability

All code used to analyze the data in this study is available from the corresponding author upon reasonable request.
