# Supplementary material for: Mfap4: a promising target for enhanced liver regeneration and chronic liver disease treatment
Source: NPJ Regen Med. 2023 Nov 7;8:63. doi: 10.1038/s41536-023-00337-9 (PMC10630300; doi:10.1038/s41536-023-00337-9)
Supplement: Supplementary file 1 — Supplementary_Material [file 41536_2023_337_MOESM1_ESM.pdf]

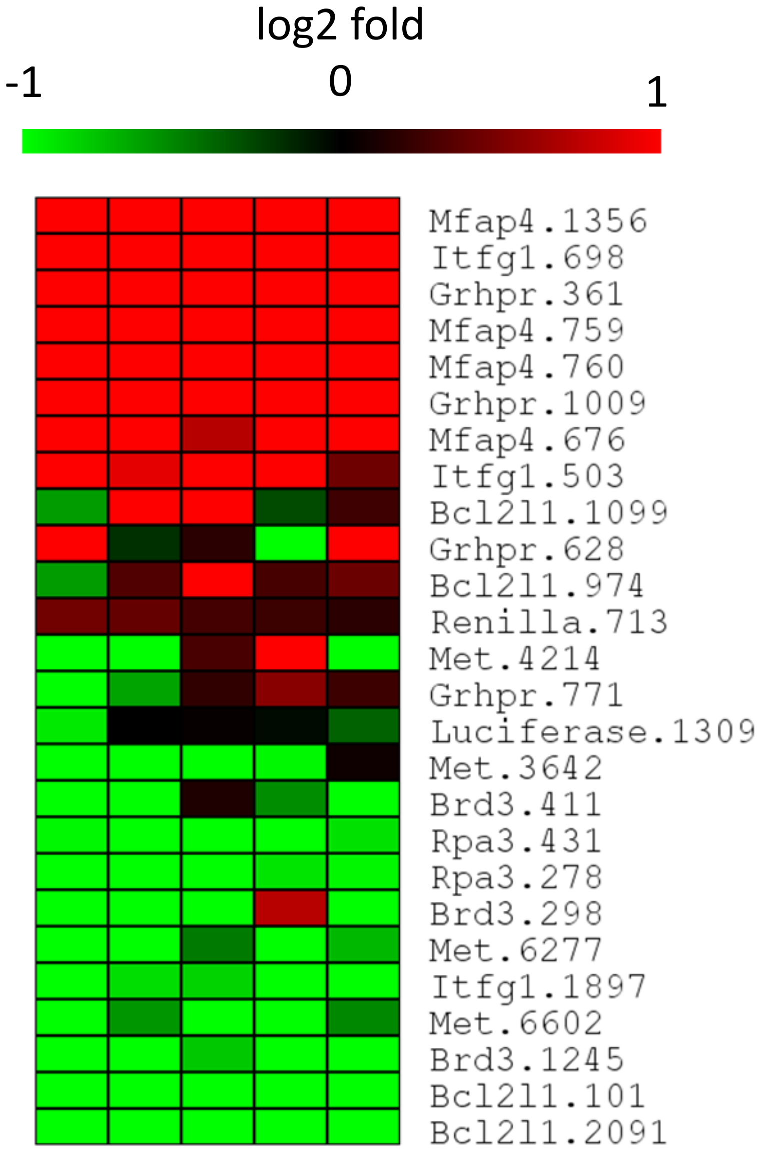

Supplementary Figure 1. Heat map of selected top-enriched and depleted shRNAs. Shown is the log2 fold change including non-targeting control shRNAs. Red are enriched candidates, and green are depleted. Each column represents one individual animal.

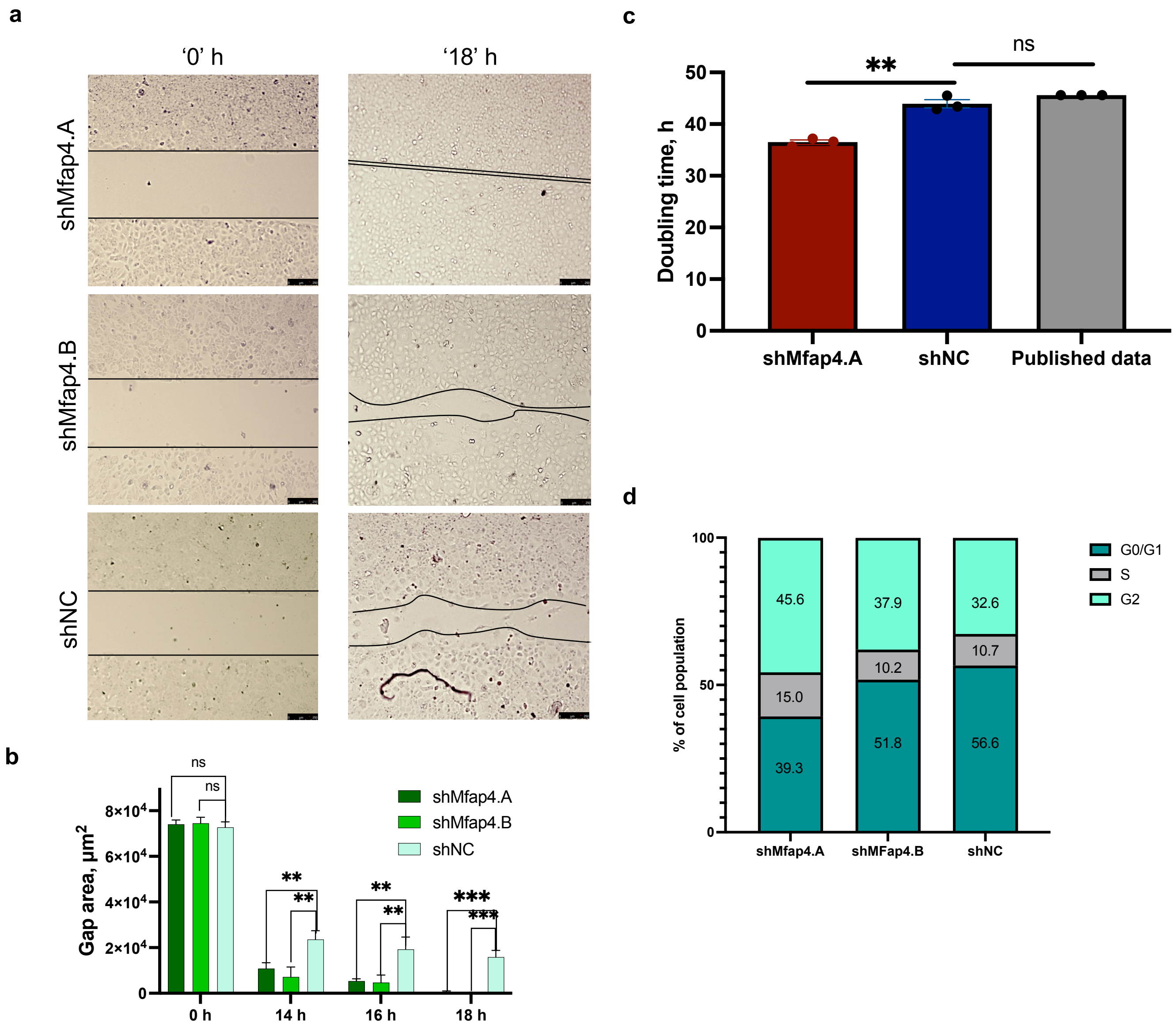

Supplementary Figure 2. shRNA mediated knockdown of *Mfap4* accelerates proliferation of liver cells. **a** Wound healing assay using AML 12 cell line with stable expression of shMfap4.A, shMfap4.B, and shNC. Cell lines were grown to full confluence, then the silicon gasket was removed leaving a defined cell free area (0h). Filling of this "wound" gap was monitored (18h) (3 technical replicates, bar = 250 μm). **b** Size of the wound area (in μm^2) ± SEM (n = 3; *p<0.05, **p<0.01,***p<0.005, ns=non - significant). **c** Shown is the doubling time for BNL.CL2 cells with stable expression of shMfap4.A, shNC, or without any shRNA. Cells were seeded at the same seeding densities. Doubling time was calculated based on the exponential phase of the growth curve. Three technical replicates were performed (published data refers to Ou, YH et al.). **d** Cell cycle analysis by flow cytometry using the Guava Muse Cell Analyzer of BNL.CL2 cells with stable expression of shMfap4.A, shMfap4.B, or shNC. Shown is the percentage of cells in the indicated cell cycle phase.

Ou, Y.H., *et al.* Butachlor, a suspected carcinogen, alters growth and transformation characteristics of mouse liver cells. *Chem Res Toxicol* **13**, 1321-1325 (2000).

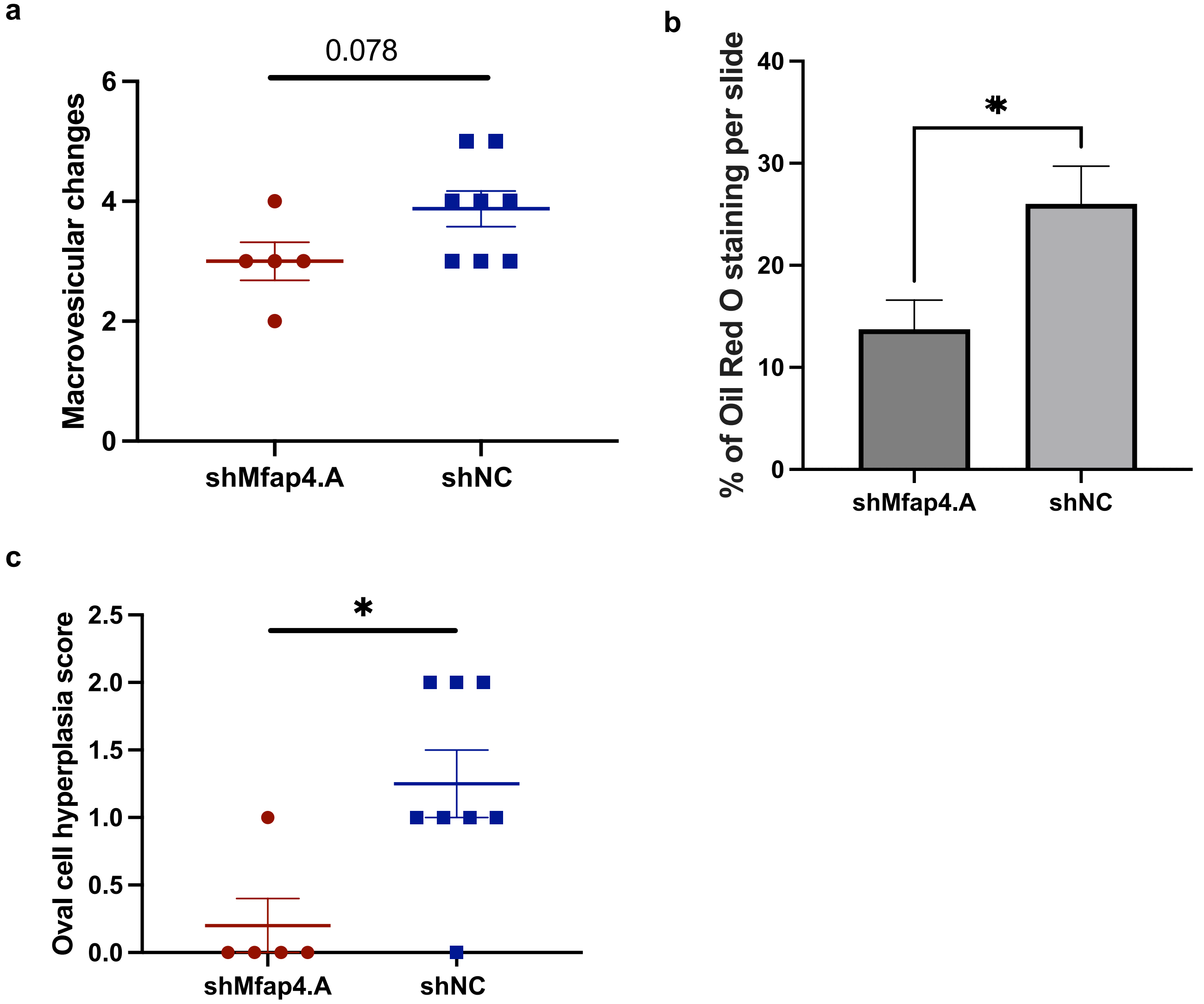

Supplementary Figure 3. shRNA medicated knockdown of *Mfap4* reduces steatosis and oval cell hyperplasia in mice exposed to the "Western" diet. a Reduction of macrovesicular changes upon *Mfap4* knockdown is detected by a certified pathologist compared to the control group (each dot represents one animal). **b** Quantification of Oil Red O stained samples. The percentage of red stained areas to the whole picture area was calculated per slide (5 areas per sample). A significant reduction of steatosis is observed (*p<0.05). **c** Significant reduction of oval cell hyperplasia in the case of *Mfap4* knockdown compared to the non-targeting control. Oval cell hyperplasia score is determined by a certified pathologist, who was blinded regarding the experimental group (each point represents one animal; *p<0.05).

a

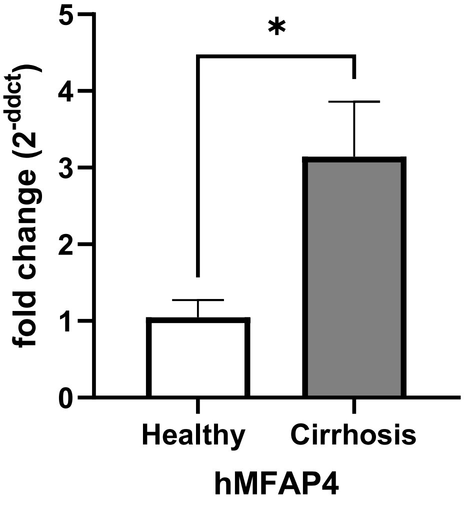

b

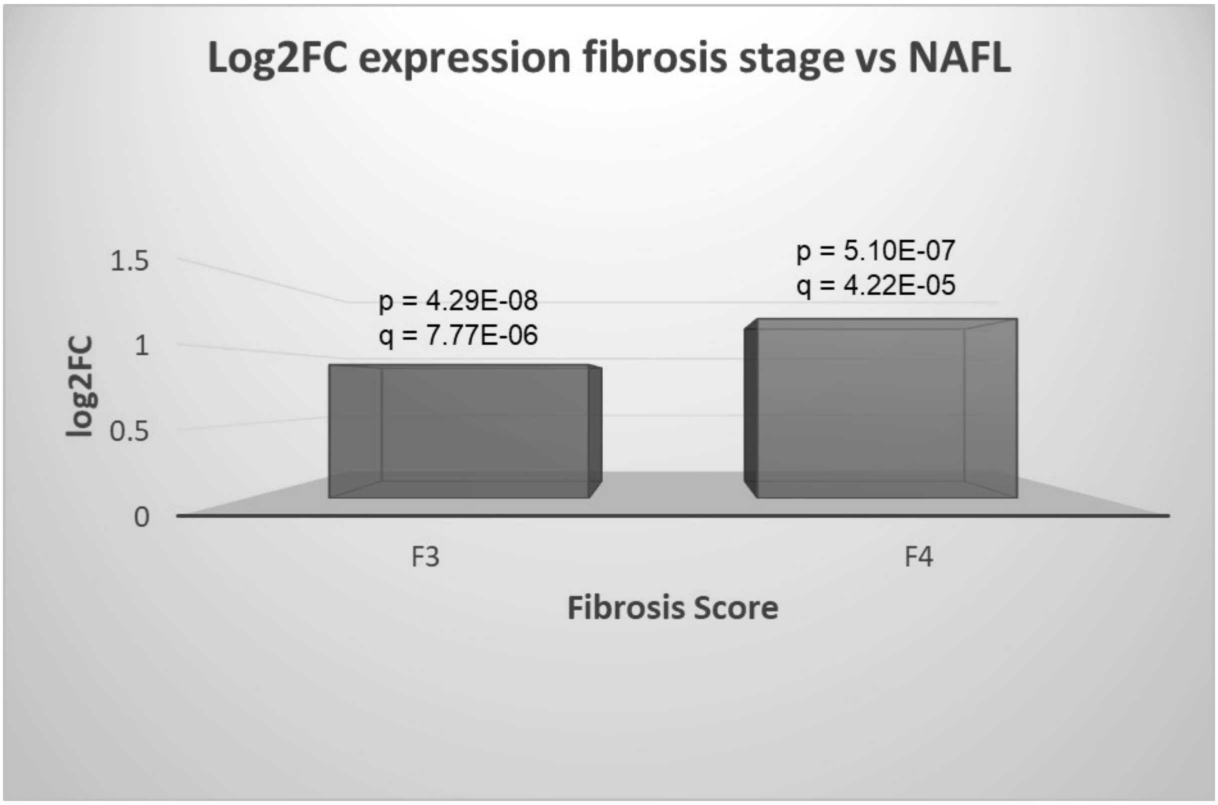

Supplementary Figure 4. *MFAP4* shows fibrosis stage dependent significant increased expression. a Higher expression of MFAP4 is detected in cirrhotic livers compared to healthy donors ($p<0.005$). **b** Based on the supplementary table provided in the Govaere et al. study of a nonalcoholic fatty liver disease patient cohort, *MFAP4* shows a highly significant upregulation at fibrosis score 3 and 4 vs simple steatosis (p-value of 4.29E-08 / q-value of 7.77E-06 and p-value of 5.10E-07 / q-value of 4.22E-05, respectively; based on the analysis of 206 patients).

Govaere, O., et al. Transcriptomic profiling across the nonalcoholic fatty liver disease spectrum reveals gene signatures for steatohepatitis and fibrosis. Sci Transl Med 12(2020).

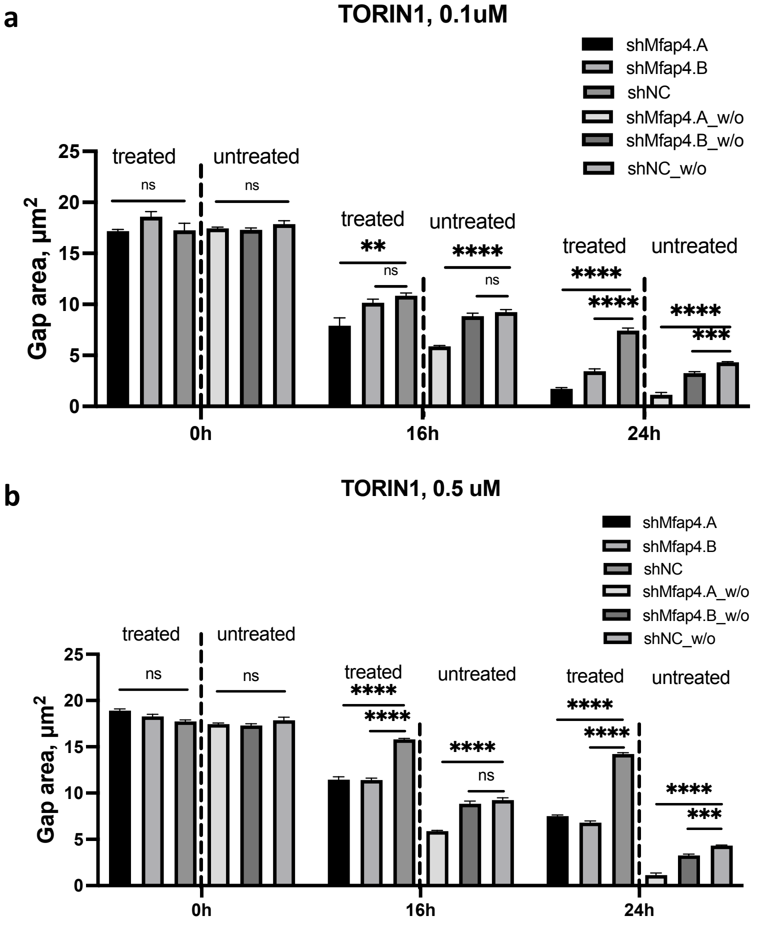

Supplementary Figure 5. Inhibition of mTOR strongly attenuates *Mfap4* knockdown driven faster wound healing. Quantification of Wound healing assay is shown. Cell lines with stable *Mfap4* knockdown were treated with the mTOR inhibitor Torin or carrier. Wound gap closure was monitored **a** 0.1μ*M* Torin1 was administered **b** 0.5 μ*M* Torin1 was administered. Data were analyzed by ImageJ software and 2-way ANOVA statistical test was performed to determine significance.

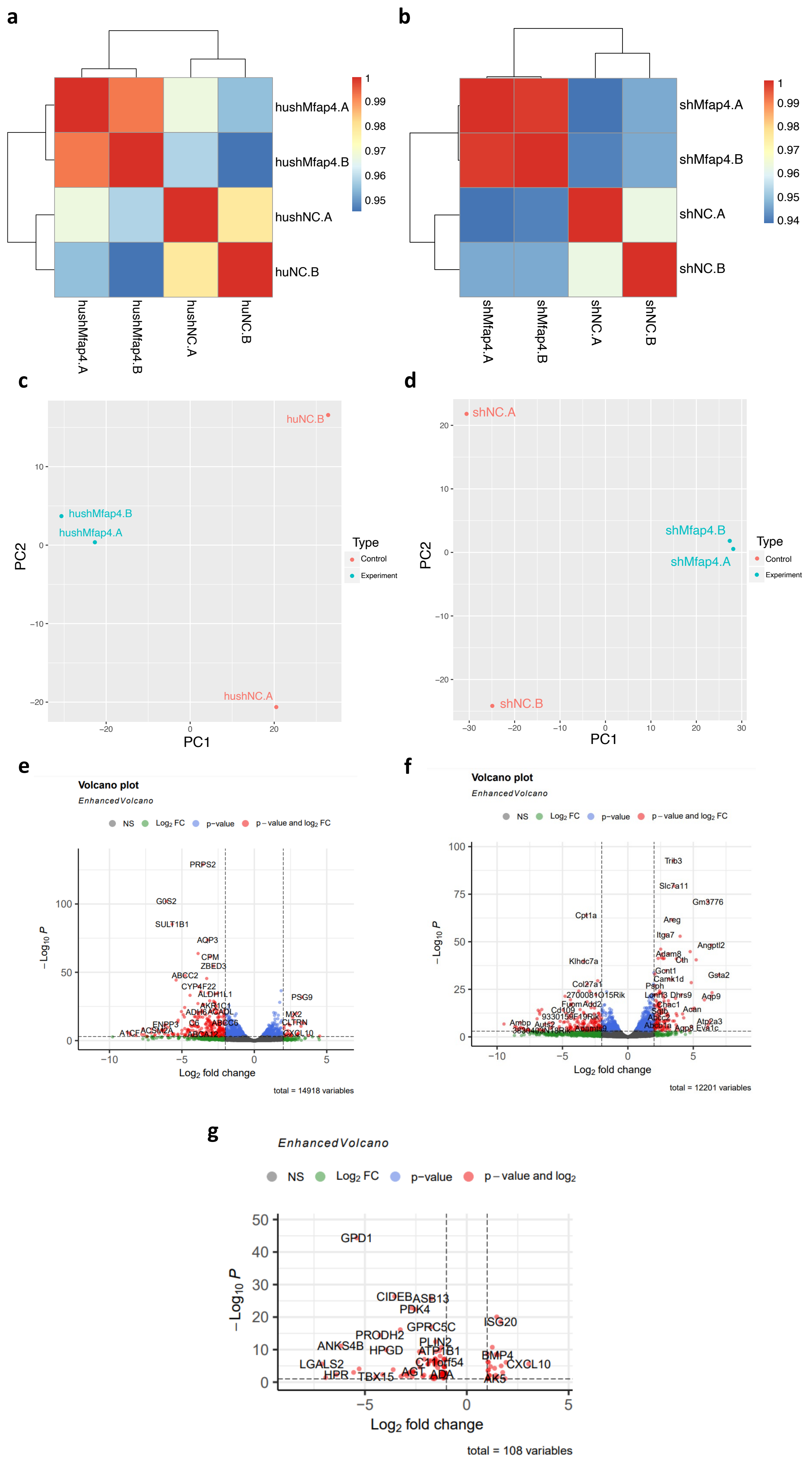

a
hushMfap4.A
hushMfap4.B
hushNC.A
huNC.B
1
0.99
0.98
0.97
0.96
0.95
b
shMfap4.A
shMfap4.B
shNC.A
shNC.B
1
0.99
0.98
0.97
0.96
0.95
0.94
c
huNC.B
hushMfap4.B
hushMfap4.A
hushNC.A
PC1
PC2
Type
Control
Experiment
d
shNC.A
shMfap4.B
shMfap4.A
shNC.B
PC1
PC2
Type
Control
Experiment
e
Volcano plot
EnhancedVolcano
NS
Log2 FC
p-value
p-value and log2 FC
PRPS2
G0S2
SULT1B1
AQP3
CPM
ZBED3
ABCC2
CYP4F22
ALDH1L1
PSG9
AKR1C1
ADH6
ACADL
MX2
CLTRN
ENPP3
C6
ABCC6
A1CF
ACSM2A
ABCA12
CXCL10
−Log10 P
Log2 fold change
total = 14918 variables
f
Volcano plot
EnhancedVolcano
NS
Log2 FC
p-value
p-value and log2 FC
Trib3
Slc7a11
Gm3776
Cpt1a
Areg
Itga7
Angptl2
Klhdc7a
Adam8
Cth
Gcnt1
Camk1d
Gsta2
Col27a1
Psph
2700081O15Rik
Lonrf3
Dhrs9
Aqp9
Fcgbp
Add2
Chac1
Cd109
Acan
9330159F19Rik
Sgcb
Abcc2
Ambp
Auts2
Abcb1a
Atp2a3
Adamts9
Aqp8
Eva1c
−Log10 P
Log2 fold change
total = 12201 variables
g
EnhancedVolcano
NS
Log2 FC
p-value
p-value and log2
GPD1
CIDEB
ASB13
PDK4
ISG20
GPRC5C
PRODH2
PLIN2
ANKS4B
HPGD
ATP1B1
BMP4
CXCL10
LGALS2
C11orf54
HPR
TBX15
AGT
ADA
AK5
−Log10 P
Log2 fold change
total = 108 variables

Supplementary Figure 6. Interspecies trancriptomic profiling of immortalized human and mouse hepatocytes with Mfap4 knockdown. a Correlation heat map for immortalized human hepatocytes with *MFAP4* knockdown and respective control. **b** Correlation map for immortalized mouse hepatocytes with *Mfap4* knockdown and respective control. **c** PCA plot for immortalized human hepatocytes with *MFAP4* knockdown and respective control. **d** PCA plot for immortalized mouse hepatocytes with *Mfap4* knockdown and respective control. **e** Volcano plot of expressed genes comparing immortalized human hepatocytes with *MFAP4* knockdown and respective control. **f** Volcano plot of expressed genes comparing immortalized mouse hepatocytes with *Mfap4* knockdown and respective control. **g** Integration of the interspecies transcriptome analysis. Volcano plot of significantly differentially expressed genes conserved between both species mouse and man.

a

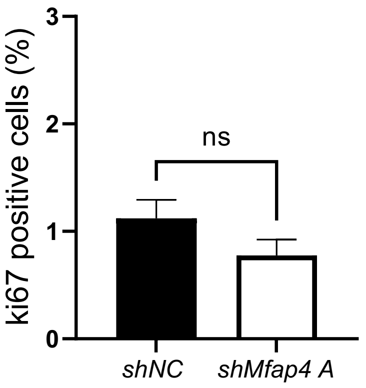

b

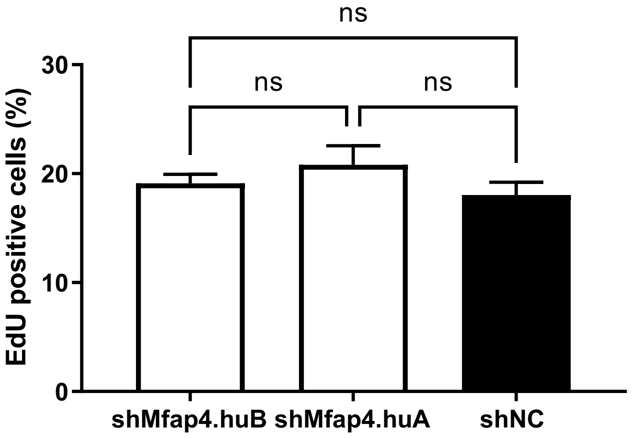

Supplementary Figure 7 Mfap4 knockdown does not drive liver cancer or unregulated proliferation. a Quantification of Ki67 positive hepatocytes. Shown are % Ki67 positive cells in mouse livers after 1 year repopulation. Data shows average ± SEM (ns=non-significant). **b** DNA synthesis of HepG2 cells with stable expression of shMfap4.A, shMfap4.B or shNC was assessed by EdU incorporation. Shown is the value of % EdU positive cells ± SEM (ns=non-significant, 3 independent replicates).

Gene name	Primer sequence Forward	Primer sequence Reverse
MFAP4	AGGAGATGCTCTGGAGAGG	AAAACCGTCCACTTCCCGCC
LRP8	GCCAAGGATTGCGAAAAGGAC	GTGGTCTAAGCAGTCATCGTC
DUSP4	AGTCTCAAGGCGGCTATGAG	AGGACCCCCCTGGTCGTG
DUSP5	TGTCGTCCTCACCTCGCTA	GGGCTCTCTCACTCTCAATCTTC
PLAT	AGCGAGCCAAGGTGTTTCAA	CTTCCCAGCAAATCCTTCGGG
SLC7A5	AGATCGGGAAGGGTGATGTG	AATTCAAGTAATTCCATCCTCC
GAPDH	TCAAGATCATCAGCAATGCC	ACTGTGGTCATGAGTCCTTC

Supplementary table 1: Primers sequences are provided.

siRNA / shRNA	Sequence
shMfap4.1356	TGCTGTTGACAGTGAGCGCCGGCTACTGCTCAACTCTGAATAGTGAAGCCACA GATGTATTCAGAGTTGAGCAGTAGCCGTTGCCTACTGCCTCGGA
shMfap4.760	TGCTGTTGACAGTGAGCGCAGGCTTCTATTACTCCCTCAATAGTGAAGCCACAG ATGTATTGAGGGAGTAATAGAAGCCTTTGCCTACTGCCTCGGA
shNC	AAGGTATATTGCTGTTGACAGTGAGCGCGCAGTTCGATATCTACTGAAATAGT GAAGCCACAGATGTATTTCAGTAGATATCGAACTGCTTGCCTACTGCCTCG
siP70S6K	Dharmacon (Horizon) SMARTpool; 72508, 5nmol
sihuMfap4	Dharmacon (Horizon) SMARTpool; 4239, 5nmol
siNC	Dharmacon (Horizon) Non-targeting SMARTpool, 5nmol
shhuMfap4. 1602	TGCTGTTGACAGTGAGCGATAGGGACTGAAGGTCTCAATATAGTGAAGCCACA GATGTATATTGAGACCTTCAGTCCCTACTGCCTACTGCCTCGGA
shNC (shRenila)	TGCTGTTGACAGTGAGCGCAGGAATTATAATGCTTATCTATAGTGAAGCCACA GATGTATAGATAAGCATTATAATTCCTATGCCTACTGCCTCGGA

Supplementary table 2: siRNA/shRNA sequences are provided.

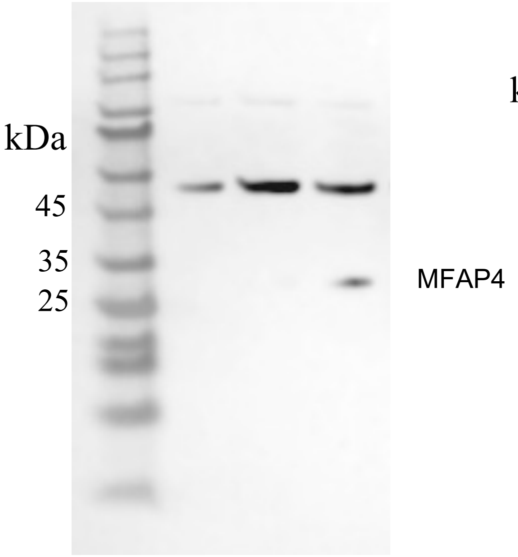

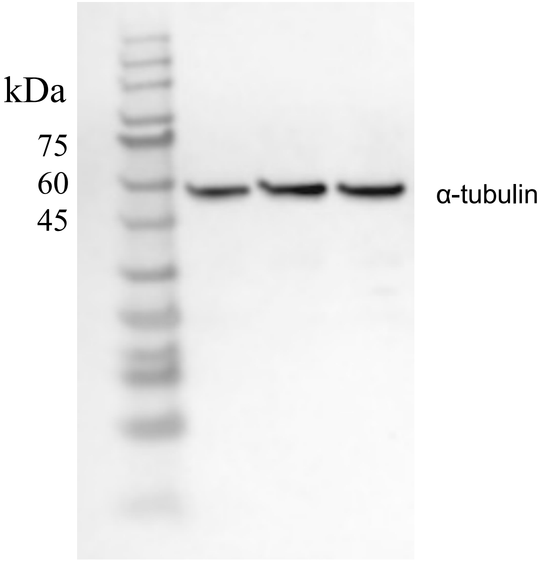

Uncropped Western Blot for Figure 2b for Mfap4.

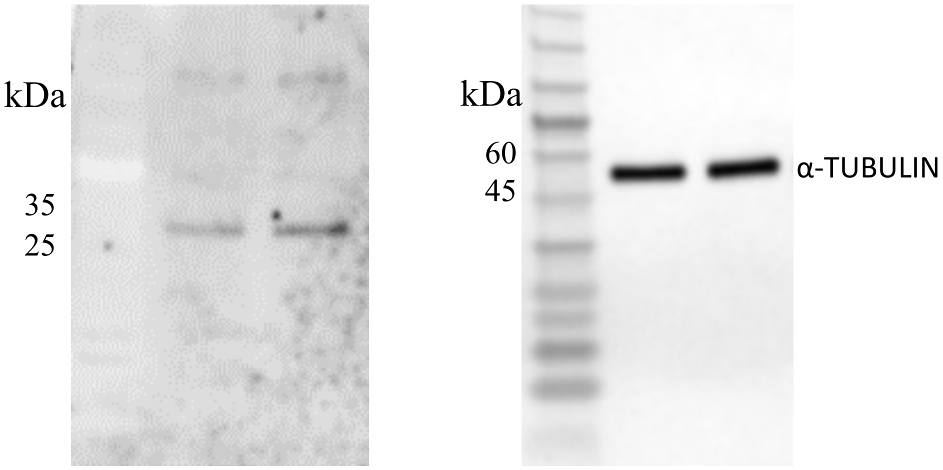

Uncropped Western Blot for Figure 5e for *si huMAFP4*.

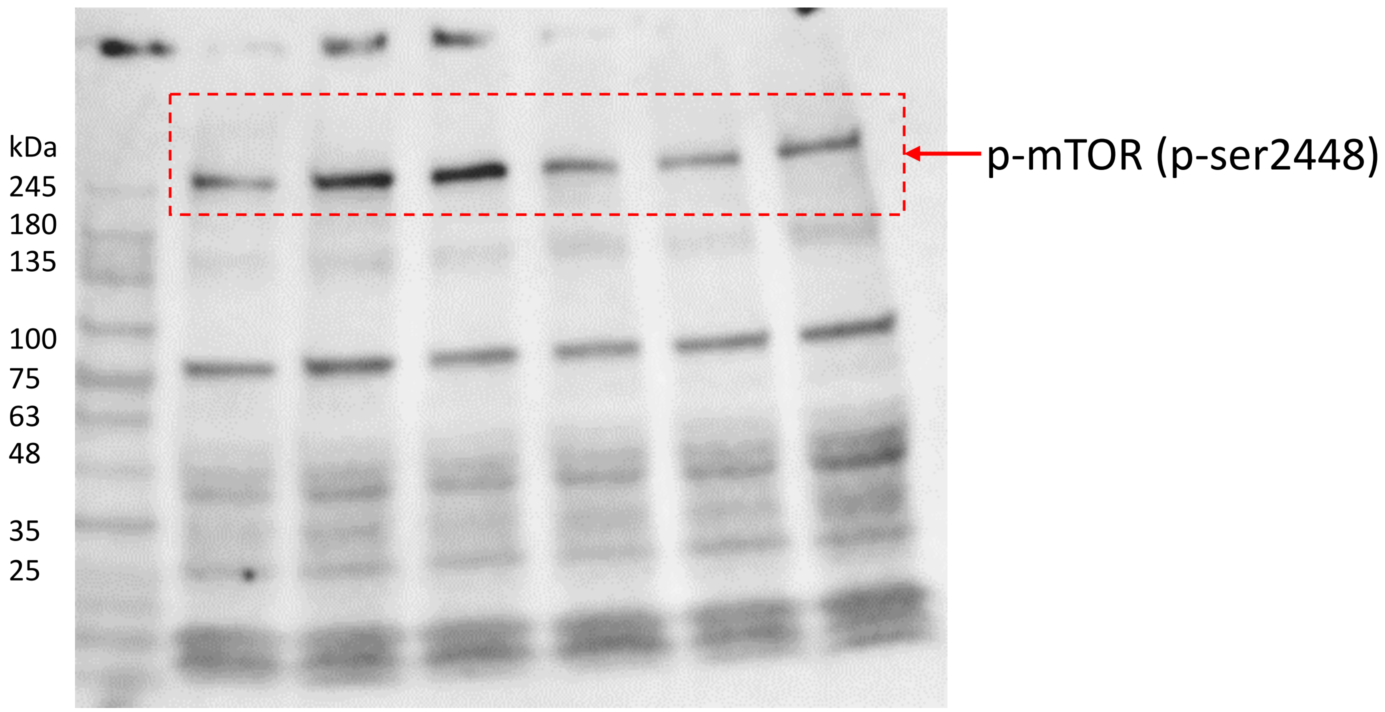

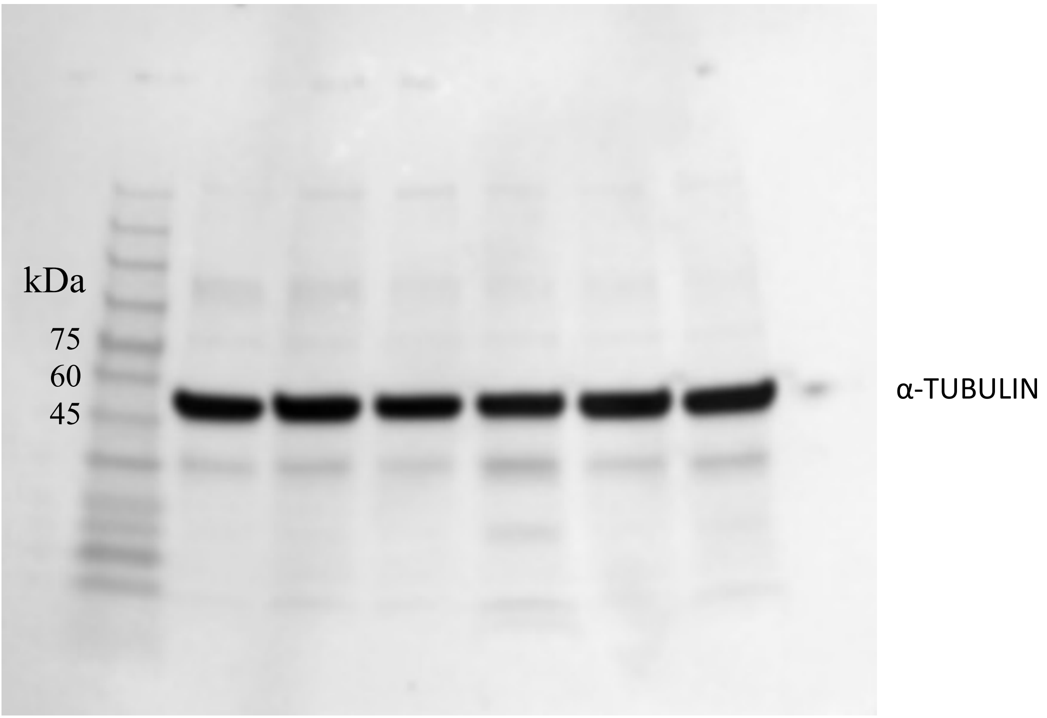

Uncropped Western Blot for Figure 6c for p-mTOR.

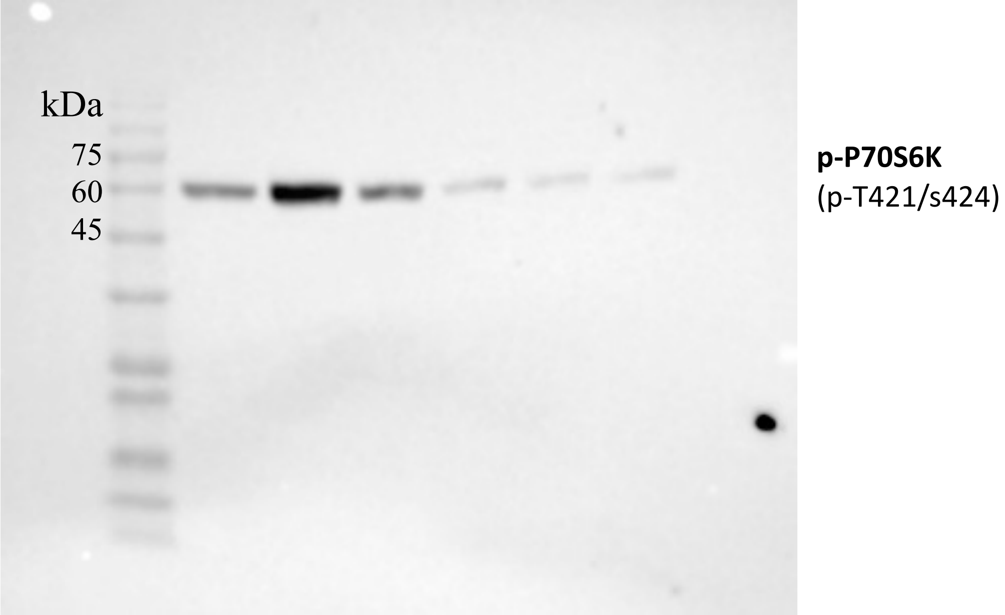

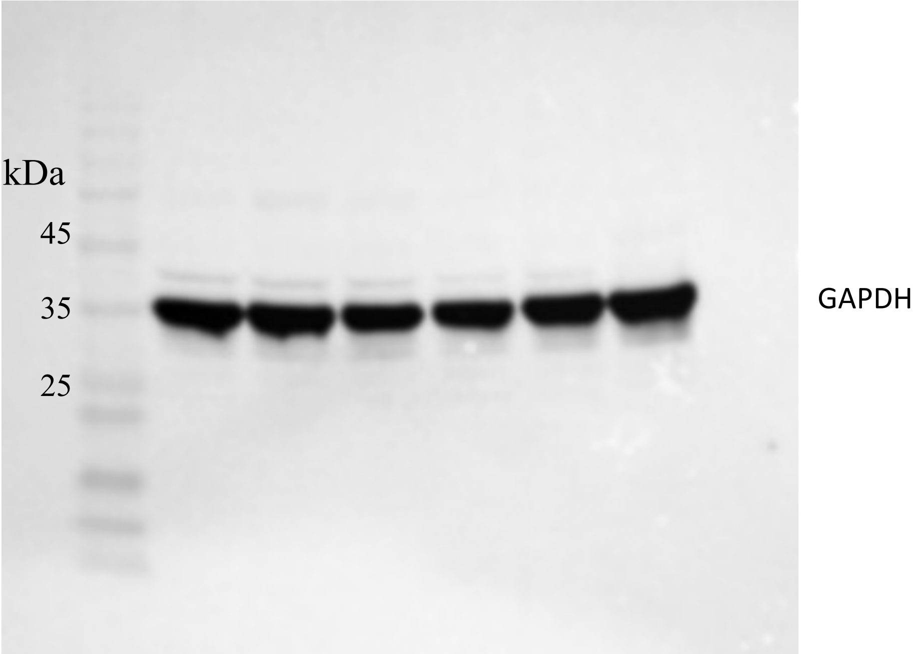

Uncropped Western Blot for Figure 6c for p-P70S6K.

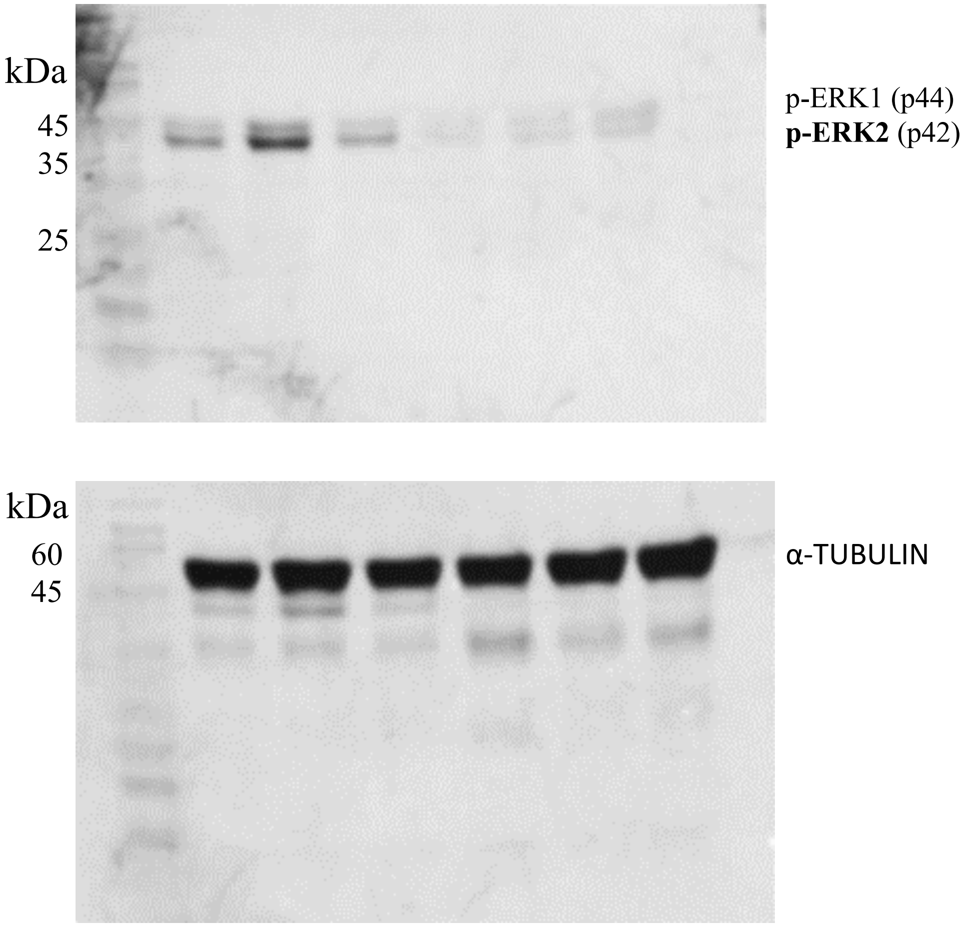

Uncropped Western Blot for Figure 6c for p-ERK.
