# Supplementary material for: Mfap4: a promising target for enhanced liver regeneration and chronic liver disease treatment
Source: NPJ Regen Med. 2023 Nov 7;8:63. doi: 10.1038/s41536-023-00337-9 (PMC10630300; doi:10.1038/s41536-023-00337-9)
Supplement: Supplementary file 2 — Reporting-summary [file 41536_2023_337_MOESM2_ESM.pdf]

nature portfolio

Corresponding author(s): Torsten Wuestefeld

Last updated by author(s): Oct 4, 2023

Reporting Summary

Nature Portfolio wishes to improve the reproducibility of the work that we publish. This form provides structure for consistency and transparency in reporting. For further information on Nature Portfolio policies, see our Editorial Policies and the Editorial Policy Checklist.

Statistics

For all statistical analyses, confirm that the following items are present in the figure legend, table legend, main text, or Methods section.

n/a	Confirmed	
☐	☒	The exact sample size (*n*) for each experimental group/condition, given as a discrete number and unit of measurement
☐	☒	A statement on whether measurements were taken from distinct samples or whether the same sample was measured repeatedly
☐	☒	The statistical test(s) used AND whether they are one- or two-sided<br>*Only common tests should be described solely by name; describe more complex techniques in the Methods section.*
☒	☐	A description of all covariates tested
☒	☐	A description of any assumptions or corrections, such as tests of normality and adjustment for multiple comparisons
☒	☐	A full description of the statistical parameters including central tendency (e.g. means) or other basic estimates (e.g. regression coefficient) AND variation (e.g. standard deviation) or associated estimates of uncertainty (e.g. confidence intervals)
☐	☒	For null hypothesis testing, the test statistic (e.g. F, t, r) with confidence intervals, effect sizes, degrees of freedom and P value noted<br>*Give P values as exact values whenever suitable.*
☒	☐	For Bayesian analysis, information on the choice of priors and Markov chain Monte Carlo settings
☒	☐	For hierarchical and complex designs, identification of the appropriate level for tests and full reporting of outcomes
☒	☐	Estimates of effect sizes (e.g. Cohen's *d*, Pearson's *r*), indicating how they were calculated

Our web collection on statistics for biologists contains articles on many of the points above.

Software and code

Policy information about availability of computer code

Data collection	Imaging data was collected using Leica Application Suite X 3.0.0.15697, GeneSys V1.5.0.0 Syngene, ImageJ NIH, Partek Genomics Suite 7.0 Partek
Data analysis	GraphPad Prism 9 was used for statistical analysis; differentially expressed genes were identified separately for human and mouse samples using the “DESeq2” R package; human orthologs of mouse genes were obtained using the “biomaRt” R package by obtaining the “hgnc symbol” corresponding to the “mgi symbol” of genes in the mouse data with getLDS() function.

For manuscripts utilizing custom algorithms or software that are central to the research but not yet described in published literature, software must be made available to editors and reviewers. We strongly encourage code deposition in a community repository (e.g. GitHub). See the Nature Portfolio guidelines for submitting code & software for further information.

Data

Policy information about availability of data

All manuscripts must include a data availability statement. This statement should provide the following information, where applicable:

- Accession codes, unique identifiers, or web links for publicly available datasets
- A description of any restrictions on data availability
- For clinical datasets or third party data, please ensure that the statement adheres to our policy

Raw data for this study were generated at Genome Institute of Singapore core facility. Count matrix file for the screen will be provided as supplementary.

Research involving human participants, their data, or biological material

Policy information about studies with human participants or human data. See also policy information about sex, gender (identity/presentation), and sexual orientation and race, ethnicity and racism.

Reporting on sex and gender	Only unidentifiable/deidentified human samples were used. Therefore no gender information is available.
Reporting on race, ethnicity, or other socially relevant groupings	Only unidentifiable/deidentified human samples were used. Therefore no such information is available.
Population characteristics	Only unidentifiable/deidentified human samples were used. Therefore no such information is available.
Recruitment	Only unidentifiable/deidentified human samples were used. Therefore no such information is available.
Ethics oversight	National University Hospital, Singapore

Note that full information on the approval of the study protocol must also be provided in the manuscript.

Field-specific reporting

Please select the one below that is the best fit for your research. If you are not sure, read the appropriate sections before making your selection.

☒ Life sciences ☐ Behavioural & social sciences ☐ Ecological, evolutionary & environmental sciences

For a reference copy of the document with all sections, see nature.com/documents/nr-reporting-summary-flat.pdf

Life sciences study design

All studies must disclose on these points even when the disclosure is negative.

Sample size	Sample size of animals constituted at least 4 mice per each group to be able to identify statistical significance (if any)
Data exclusions	No data were excluded from the analyses
Replication	All attempts of technical and/or biological replications were successful
Randomization	Samples/ animals/ participants were allocated randomly for this study
Blinding	Pathologist was blinded regarding sample evaluation.

Reporting for specific materials, systems and methods

We require information from authors about some types of materials, experimental systems and methods used in many studies. Here, indicate whether each material, system or method listed is relevant to your study. If you are not sure if a list item applies to your research, read the appropriate section before selecting a response.

Materials & experimental systems

n/a	Involved in the study	
☐	☒	Antibodies
☐	☒	Eukaryotic cell lines
☒	☐	Palaeontology and archaeology
☐	☒	Animals and other organisms
☒	☐	Clinical data
☒	☐	Dual use research of concern
☒	☐	Plants

Methods

n/a	Involved in the study	
☒	☐	ChIP-seq
☒	☐	Flow cytometry
☒	☐	MRI-based neuroimaging

Antibodies

Antibodies used	MFAP4 Polyclonal Antibody (ThermoFisher Scientific, Cat# PA5-42013; RRID:AB_2608506), Anti-Ki67 antibody (Abcam, Ca# ab15580; RRID:AB_443209), GFP antibody (Cell Signaling Technology, Cat# 2956; RRID:AB_1196615), Phospho-p70S6 Kinase (Thr421/Ser424) Antibody (Cell Signaling Technology, Cat# 9204; RRID:AB_2265913), P70S6 Kinase (Cell Signaling Technology, Cat# 2708), Phospho-mTOR (Ser2448) Antibody (Cell Signaling Technology, Cat# 2971;RRID:AB_330970), a-Tubulin (Cell Signaling Technology, Cat# 2125),

	Rabbit Anti-GAPDH Monoclonal Antibody (Cell Signaling Technology, Cat# 2118; RRID:AB_561053), Anti-Rabbit IgG (H+L), HRP Conjugate (Promega, Cat# W4011, RRID:AB_430833), Anti-Mouse IgG (H+L), HRP Conjugate (Promega, Cat# W4021, RRID:AB_430834), p-SAPK/JNK (Cell Signaling Technology, Cat# 9251), MKK6 (Cell Signaling Technology, Cat# 8550), p-RPS6 (Cat#2211), RPS6 (Cell Signaling Technology, Cat# 2217), p-MKK3/MKK6 (Cell Signaling Technology, Cat# 12280), p-ERK1/ERK2 (Cell Signaling Technology, Cat# 4370).
Validation	All antibody were first validated using proteins isolated from the mouse or human cell lines according to the protocol provided by vendor with an antibody

Eukaryotic cell lines

Policy information about cell lines and Sex and Gender in Research

Cell line source(s)	Phoenix-AMPHO packaging cells (ATCC, Cat # CRL-3213; RRID:CVCL_H716), BNL CL.2 cell line (TIB 73) (ATCCCat# CRL-3308; RRID:CVCL_JM59), AML 12 cell line (ATCC, Cat# CRL-2254, RRID:CVCL_0140), Immortalized Human Hepatocytes SV40 (Creative Bioarray Cat # CSC-I9016L)
Authentication	Ordered from ATCC (https://www.atcc.org/), Creative Bioarray (https://www.creative-bioarray.com)
Mycoplasma contamination	All cell lines were tested negative for Mycoplasma contamination
Commonly misidentified lines (See ICLAC register)	None of misidentified cell lines were used

Animals and other research organisms

Policy information about studies involving animals; ARRIVE guidelines recommended for reporting animal research, and Sex and Gender in Research

Laboratory animals	C57BL/6JInv 5 weeks old female and male mice (IMSR_JAX:000664), C.B-17 SCID 5 weeks old female and male mice (C.B-Igh-1b/IcrTac-Prkdcscid). Mice were ordered from InVivos (https://www.invivos.com.sg/). FAH knockout mice have been generated by Dr. Markus Grompe (Oregon Health Sciences University) and were obtained in the C57BL/6 background from Dr. Arndt Vogel (Hannover Medical School)
Wild animals	n/a
Reporting on sex	The study is sex independent. Sex data was provided by the vendor (InVivos (https://www.invivos.com.sg/). Mice were ordered mix gender in order to perform sex independent study.
Field-collected samples	n/a
Ethics oversight	All mice were housed and maintained under pathogen free conditions in accordance with the institutional guidelines of the Biological Resource Centre (BRC), A*STAR, Singapore. All animal experiments have been approved by the Institutional Animal Care and Use Committee (A*Star, IACUC No. 191452, Singapore)

Note that full information on the approval of the study protocol must also be provided in the manuscript.
